# SAR-mediated Similarity Assessment of the Property Profile for New, Silicon-Based AChE/BChE Inhibitors

**DOI:** 10.3390/ijms20215385

**Published:** 2019-10-29

**Authors:** Andrzej Bak, Hana Pizova, Violetta Kozik, Katarina Vorcakova, Jiri Kos, Jakub Treml, Klara Odehnalova, Michal Oravec, Ales Imramovsky, Pavel Bobal, Adam Smolinski, Zdeněk Trávníček, Josef Jampilek

**Affiliations:** 1 Institute of Chemistry, University of Silesia, Szkolna 9, 40 007 Katowice, Poland; violetta.kozik@us.edu.pl; 2 Department of Chemical Drugs, Faculty of Pharmacy, University of Veterinary and Pharmaceutical Sciences, Palackeho 1, 612 42 Brno, Czech Republic; odehnalovak@vfu.cz (K.O.); pavelbobal@yahoo.com (P.B.); 3 Department of Biological and Biochemical Sciences, Faculty of Chemical Technology, University of Pardubice, Studentska 573, 532 10 Pardubice, Czech Republic; katarina.vorcakova@upce.cz; 4 Division of Biologically Active Complexes and Molecular Magnets, Regional Centre of Advanced Technologies and Materials, Faculty of Science, Palacky University, Slechtitelu 27, 783 71 Olomouc, Czech Republic, jiri.kos@upol.cz (J.K.); zdenek.travnicek@upol.cz (Z.T.); 5 Department of Molecular Biology and Pharmaceutical Biotechnology, Faculty of Pharmacy, University of Veterinary and Pharmaceutical Sciences, Palackeho 1, 612 42 Brno, Czech Republic; tremlj@vfu.cz; 6 Global Change Research Institute CAS, Belidla 986/4a, 60300 Brno, Czech Republic; oravec.m@czechglobe.cz; 7 Institute of Organic Chemistry and Technology, Faculty of Chemical Technology, University of Pardubice, Studentska 573, 532 10 Pardubice, Czech Republic; ales.imramovsky@upce.cz; 8 Department of Energy Saving and Air Protection, Central Mining Institute, Plac Gwarkow 1, 40 166 Katowice, Poland; smolin@gig.katowice.pl

**Keywords:** silicon-based carbamates, in vitro cholinesterase inhibition, CoMSA, IVE-PLS, molecular docking, similarity-activity landscape index

## Abstract

A set of 25 novel, silicon-based carbamate derivatives as potential acetyl- and butyrylcholinesterase (AChE/BChE) inhibitors was synthesized and characterized by their in vitro inhibition profiles and the selectivity indexes (SIs). The prepared compounds were also tested for their inhibition potential on photosynthetic electron transport (PET) in spinach (*Spinacia oleracea L.*) chloroplasts. In fact, some of the newly prepared molecules revealed comparable or even better inhibitory activities compared to the marketed drugs (rivastigmine or galanthamine) and commercially applied pesticide Diuron^®^, respectively. Generally, most compounds exhibited better inhibition potency towards AChE; however, a wider activity span was observed for BChE. Notably, benzyl *N*-[(*1S*)-2-[(*tert*-butyldimethylsilyl)oxy]-1-[(2-hydroxyphenyl)carbamoyl]ethyl]-carbamate (**2**) and benzyl *N*-[(*1S*)-2-[(*tert*-butyldimethylsilyl)oxy]-1-[(3-hydroxyphenyl)carbamoyl]ethyl]-carbamate (**3**) were characterized by fairly high selective indexes. Specifically, compound **2** was prescribed with the lowest IC_50_ value that corresponds quite well with galanthamine inhibition activity, while the inhibitory profiles of molecules **3** and benzyl-*N*-[(*1S*)-2-[(*tert*-butyldimethylsilyl)oxy]-1-[(4-hydroxyphenyl)carbamoyl]ethyl]carbamate (**4**) are in line with rivastigmine activity. Moreover, a structure–activity relationship (SAR)-driven similarity evaluation of the physicochemical properties for the carbamates examined appeared to have foreseen the activity cliffs using a similarity–activity landscape index for BChE inhibitory response values. The ‘indirect’ ligand-based and ‘direct’ protein-mediated in silico approaches were applied to specify electronic/steric/lipophilic factors that are potentially valid for quantitative (Q)SAR modeling of the carbamate analogues. The stochastic model validation was used to generate an ‘average’ 3D-QSAR pharmacophore pattern. Finally, the target-oriented molecular docking was employed to (re)arrange the spatial distribution of the ligand property space for BChE and photosystem II (PSII).

## 1. Introduction

Finding critical in vitro and/or in silico parameters (descriptors or properties) of the hit→lead→seed→drug route is still a long-term aspiration and monumental challenge in drug discovery projects, mainly due to the intrinsic multidimensionality of the problem [1]. In concert with the medicinal chemist’s intuition (or serendipity), the computer-assisted molecular design (CAMD) approaches are regarded as an integral method for the exhaustive transformation of the compound topology or topography and/or the electronic properties into the property-based chemical space [2]. The systematic observational of trends of structural chemical modifications that produce corresponding changes in the biological responses for a congeneric series of molecules are fundamental for the multidimensional quantitative structure–activity (mD-QSAR) studies [3]. Moving from complex biological relationships to simple QSAR models seems to be a ‘triumph of hope over experience,’ since modelers sometimes are playing a ‘glass bead game.’ The quantitative mapping of experimental/calculated properties/descriptors into the ADMET-driven molecular potency is frequently a ‘hit-or-miss affair.’ Therefore, some structure–activity relationship (SAR)-mediated guides are urgently needed at the ‘pre-synthesis’ stage [4]. 

Molecular similarity is unarguably at the core of many SAR-based methods, assuming that small variations in structure lead to small changes in activity. In other words, the working tenet of QSAR is that molecules with high similarity share common effects (similarity principle); however, the validity of the assumption is still questionable—there is no absolute standard (or metric) of similarity [5]. In fact, similarity is a subjective concept directly related to aspects of human cognition; this phenomena is sometimes called ‘psychological proximity’ [6]. Maggiora’s provocative statement that, “Similarity, like pornography, is difficult to define, but you know it when you see it,” is probably a better illustration of cognitive aspects of similarity [7]. Despite the presence of fluid scientific boundaries, e.g., some similarity metrics exhibit size-dependent behavior—similarity quantification is still a powerful concept that allows us to specify (with some confidence) the shared host–quest interactions. In fact, the idea of specifying a numerical measure of the intermolecular similarity based on the degree of the resemblance between two objects, each described by a number of attributes, has been widely applied in chemical information systems. A set of characteristics might be denoted by a bit-string representation, sometimes augmented with the weighting factors, assigning varying importance to the particular descriptors to calculate a quantitative measure of the structural resemblance between objects, for instance Tanimoto coefficient [8]. Obviously, a crucial question that confronts drug ‘hunters’ is, which properties or features should be employed in the receptor-dependent (RD) methods that rely on the principle of complementarity or in the receptor-independent (RI) ones that are mainly based on the principle of similarity? Hence, many attempts have been undertaken in order to implement some rough guidelines, sometimes called ‘rules of thumb’ for both the ligand and structure-based in silico protocols; however, even meeting the rules does not guarantee the final success of drug discovery projects [9].

A confusing aspect of many SAR analyses is that the ‘fragile event’ can occur, when even small structural changes (colloquially termed ‘magic methyl’) can result in a boost of potency for the target, or in a more detrimental manner, can completely destroy biological response—a phenomenon known as activity ‘hotspot’ or activity ‘cliff’ in structure-activity landscape [10]. In other words, finding the optimal balance between ADMET-related properties and desired drug potency (‘sweet spot’) can be rationalized graphically by extension of the 2D similarity-based projection with activity data in the form of a ‘biological response surface’ or SAR landscapes [11]. Intuitively, we feel that the structure–activity landscapes are heterogeneous in nature with unevenly distributed regions that are reminiscent of Tolkien’s Misty Mountains with gently slopes, steep cliffs and stony bridges [12]. Geographically, a successful voyage (or exploration) requires a map indicating smooth and flat regions that are easy to navigate or jagged parts that are tough to traverse. Chemically, gently sloped hills pinpoint areas where gradual structural variations are accompanied by moderate changes in activity, whereas in flat SAR landscapes, the biological data varies little over the wide modifications in the compound structure—further substitutions are not likely to result in more potent molecules. In fact, disallowed structural modifications can be highlighted in the sharply non-uniform regions where chemically related compounds are characterized by huge response variations, forming steep cliffs in the activity landscape. Moreover, chemical bridges can be observed with molecules connecting different local SAR models [13]. The detection of the activity hotspots provides reasonable hints to the key question of medicinal chemistry regarding the impact of molecule rearrangement on the activity profile. The numerical quantification of activity cliffs is frequently performed using a variety of fingerprint representations or similarity metrics; for instance, the structure–activity landscape index (SALI) has been proposed to evaluate SAR smoothness [14].

Differently substituted amide (-CONH-) or carbamate motifs (-OCONH-) are regarded as privileged structural fragments in a vast range of marketed drugs [15] and pesticides [16,17], that are capable of interacting with multiple targets (polypharmacology concept) to modulate a biological response [18,19,20,21]. Obviously, polypharmacology also implies various side-effects, as was noticed by an anonymous reviewer [22]; however, carbamate or amide-like molecules are attractive research aims to be studied by medicinal chemists; for instance, as potential cholinesterase inhibitors (ChEIs) that are used in Alzheimer’s disease (AD) pharmacotherapy [23,24,25]. Dysfunction in cognition and memory loss (dementia) are characteristic symptoms of this progressive degeneration of the central nervous system (CNS). According to the cholinergic hypothesis, a low level of acetylcholine (ACh) is postulated to be one of the crucial factors in the pathogenesis of AD; therefore, ChEIs can have a symptomatic effect in AD [26,27]. Acetylcholinesterase (AChE) and butyrylcholinesterase (BChE) are serine hydrolases that catalyze Ach’s hydrolysis in the cholinergic synapses. The higher hydrolytic activity of AChE is partly compensated by the increased or constant level of BChE during the course of Alzheimer’s disease [28]. The second generation of competitive (e.g., galanthamine, rivastigmine) or non-competitive (e.g., tacrine and donepezil) ChEIs are clinically used to reduce AD symptoms [29]. Moreover, novel anti-AD agents have been designed, revealing some central and peripheral side effects; therefore, new targets are being scrutinized extensively [30,31,32,33,34,35]. The drugs for the treatment of AD must overcome the blood–brain barrier (BBB), a serious obstacle for the permeation of drugs that require the CNS action. However, the rapid development of various nanosystems employed for nano-based drug delivery systems has great potential to facilitate the movement of drugs across all barriers, and thus, these drug carriers have been extensively studied within a strategy of direct drug delivery to the CNS [29,36].

The photosystem II core complex (PSIIcc) is a light-driven water-plastoquinone-oxidoreductase that is harbored in the thylakoid membrane. It is the primary electron donor of the photosynthetic electron transport (PET) due to its unique property of abstracting electrons from water molecules [37,38]. Over 50% of commercially available herbicides that belong to different chemical chemotypes (e.g., urea, triazine, or phenol derivatives) act by reversible binding to the reaction center (RC) of the PSII complex in order to compete with the native plastoquinone (PQ) molecules Q_A_ and Q_B_. Strictly speaking, herbicides that target PSII and inhibit PET compete with the electron acceptor PQ for binding at the Q_A_ site in the subunit D1 in order to block the electron transfer from Q_A_ to Q_B_ [39,40]. The most potent PET inhibitors contain an amide and/or carbamate fragment that can be substituted with mutual displacement of Q_B_ from the binding pocket in the D1 subunit; therefore, the effects of differently substituted amides/carbamates as anti-invasive agents that reveal the herbicidal properties are being investigated thoroughly [41,42,43]. Obviously, the clarification of the specific herbicide–amino acid residue interactions is crucial for the proper understanding of the molecular mechanism of inhibition.

In the current paper, the synthesis and SAR-driven similarity evaluation of physicochemical properties for a novel series of 25 silicon-based carbamates as potential AChE/BChE/PET inhibitors is reported. Moreover, a SAR-driven similarity evaluation of physicochemical properties for the carbamates examined is reported to foresee the activity cliffs using a similarity–activity landscape index for BChE inhibitory response values. The ‘indirect’ ligand-based and ‘direct’ protein-mediated in silico approaches were applied to specify electronic/steric/lipophilic factors that were potentially valid for the (Q)SAR modeling of the investigated carbamate analogues. The stochastic model validation (SMV) procedure was used to generate an ‘average’ CoMSA pharmacophore pattern [44]. Moreover, the target-oriented molecular docking was employed to (re)arrange the spatial distribution of the ligand property space for BChE and PSII, respectively. As a matter of fact, a consensus methodology as the combination of the pharmacophore mapping with target-tailored procedures was proposed in the investigation of the multifaceted carbamate-enzyme interactions.

## 2. Results and Discussion

### 2.1. Design and Synthesis

**Scheme 1:**
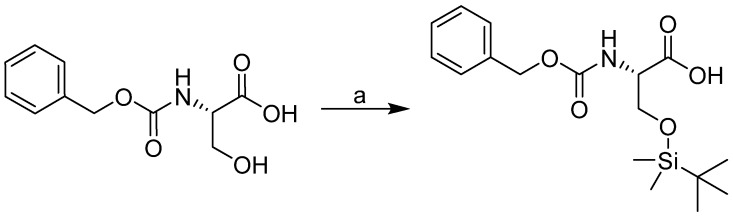
Synthesis of (2*S*)-2-{[(benzyloxy)carbonyl]amino}-3-[(*tert-*butyldimethylsilyl)oxy]- propanoic acid. Reagents and conditions: (**a**) TBDMSCl, imidazole, DMF.

The synthesis of compounds **1–25** consists of two steps. In the first step, silylation with *tert*-butyldimethylsilyl chloride (TBDMSCl) and imidazole in DMF was conducted according to literature, resulting in good yields (Scheme 1) [45].

**Scheme 2:**
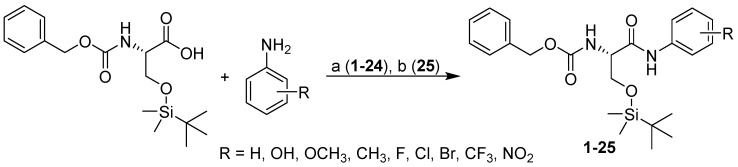
Synthesis of benzyl *N*-[(1*S*)-2-[(*tert*-butyldimethylsilyl)oxy]-1-[arylcarbamoyl]-ethyl]carbamate. Reagents and conditions: (**a**) Ethyl chloroformate, TEA and THF (**1**–**24**); (**b**) POCl_3_, pyridine (**25**).

In the second step involving amide bond formation, several chemical approaches were tested. The only known amide from the series **1–25** was compound **25** with a nitro group in position 4 of the aromatic ring [46,47,48]. In the original procedure for the synthesis of compound **25,** POCl_3_ in pyridine was used. Only amide **25** was prepared to satisfactory yield since the attempts to apply this method for the synthesis of other amide derivatives were unsuccessful [49,50]. The major problem in these syntheses seems to be desilylation and subsequent uncontrolled coupling reactions. Finally, ethyl chloroformate in the presence of triethylamine was used as a coupling reagent (see Scheme 2) [23]. Desired amides were isolated in good yields and sufficient purities.

### 2.2. Lipophilicity Measurement

The lipophilicity indexes (log *k*_w_) were measured by means of RP-HPLC method and their values are given in Table 1. The correlation coefficients of linear regression were greater than 0.98 with covariances equal or less than 0.2 units (*n* = 2). As reported in Table 1, the lipophilicity increases in order from hydroxy-substituted to trifluoromethyl-substituted derivatives.

### 2.3. In Vitro Evaluation of AChE and BChE-Inhibitory Profiles

The in vitro assessment of AChE/BChE and PET inhibitory profiles for the investigated pool of compounds was conducted and compared with marketed drugs; e.g., rivastigmine (RIV, Exelon®, Chicago, Illinois, USA), galanthamine (GLT,) and the commercially used herbicide 3-(3,4-dichlorophenyl)-1,1-dimethylurea (DCMU). Despite different mechanisms of action, RIV and GLT are the 2nd generation of ChEIs that pseudo-irreversibly inhibit AChE and BChE [51], respectively. DCMU is a sensitive and specific inhibitor of photosynthesis [52]. Table 1 reports the IC_50_ values [μM] that express quantitative measure of inhibitor concentration needed to reduce the biological process by 50%.

As illustrated in Table 1 (in bold) several of the newly synthesized molecules revealed comparable or even better inhibitory activities compared to marketed drugs (RIV and GLT) and herbicide (DCMU), respectively. Generally, more compounds exhibited a better inhibition potency towards AChE; however, a wider activity span (maximal value minus minimal one) was observed for BChE (ΔIC_50_(AChE) = 36.3 versus ΔIC_50_(BChE) = 188.63 μM). Moreover, some of compounds proved to be highly selective for BChE with respect to AChE. Notably, molecules **2** and **3** are characterized by fairly high selective indices (SI = 5.04 and SI = 3.13). Specifically, compound **2** revealed the lowest IC_50_ value (IC_50_ = 8.37 μM) that corresponds quite well with GLT inhibition activity (IC_50_ = 7.96 μM), while the inhibitory profiles of molecules **3** and **4** (IC_50_ = 19.08 and IC_50_ = 22.70 μM) are in line with RIV activity (IC_50_ = 19.95 μM).

The selectivity index of the tested compounds is not as high as for some other inhibitors investigated before. For example, Liston et al. proved that donepezil is a more than two hundred times better inhibitor of AChE than of BChE. On the other hand, tacrine is only about four times better at the inhibition of AChE [53]. However, there have been published several studies describing the less significant selectivity of carbamate inhibitors towards AChE or BChE [24,54,55,56,57]. As mentioned above, reaching the site of action is key for drugs; in the case of anti-AD drugs, this is penetration into the CNS via the BBB. A crucial step in the design of new drugs is the evaluation of their ADME/Tox profiles, frequently using predictive computational models. For all effective BChE and AChE inhibitors (compounds **2**–**4**, **25**, **7** and **6**), permeation via the BBB using ACD/Percepta 14.0.0 was predicted and compared to clinically-used drug profiles (see Tables S1–S10) and for all newly designed molecules, brain penetration sufficient for CNS activity was predicted as well.

### 2.4. Similarity-Based Assessment of Property Profiles

Molecular similarity is commonly used for estimation of the applicability domain of compounds being analyzed. The similarity-mediated evaluation of property profiles was conducted using the PCA procedure on the pool of 2774 descriptors provided by Dragon 6.0 program. The data arranged in the X_25×2774_ matrix with columns that represent parameters (variables) and rows molecules (objects) were centered and standardized. The first four principal components (PCs) characterized 72.02% of the total variable variance, while the first two PCs described 56.70%. In fact, the efficiency of data compression in PCA method is strongly dependent on the number of uncorrelated variables. In multi-dimensional data, a high percentage of variance described by the first few principal components suggests that the variables are highly inter-correlated. Conversely, smaller values of total variance characterized by the first PCs indicates a higher number of independent descriptors. To illustrate any meaningful similarity variations within the group of the investigated carbamate analogues, the projection of the chosen properties on the plane defined by the first two PCs with respect to their chemotype was carried out.

**Figure 1:**
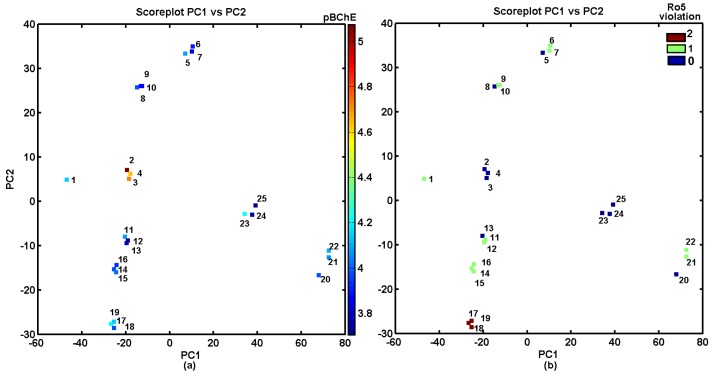
Projection of carbamates **1**–**25** on the plane defined by the first versus second principal components for Dragon descriptors with BChE activity on the logarithmic scale (**a**) and rule of five violations (**b**). Colors indicate the numerical values of pBChE activity and rule of five (Ro5) violations.

The silicon-based carbamate derivatives **1**–**25** can be classified into structurally-related groups along the first principal component revealing, basically, two clusters: the first one (PC1 > 20) that contains compounds **20**–**25** and the second one (PC1 < 20) with molecules **1**–**19** (see Figure 1a). The most potent BChE inhibitors **2**–**4** and unsubstituted molecule **1** create one group along the second principal component (0 < PC2 < 10), as shown in Figure 1a. Interestingly, the structurally related molecules **2**–**4** (the positional isomers) do not violate the Lipinski’s rule of five (Ro5), as illustrated in Figure 1b.

**Figure 2:**
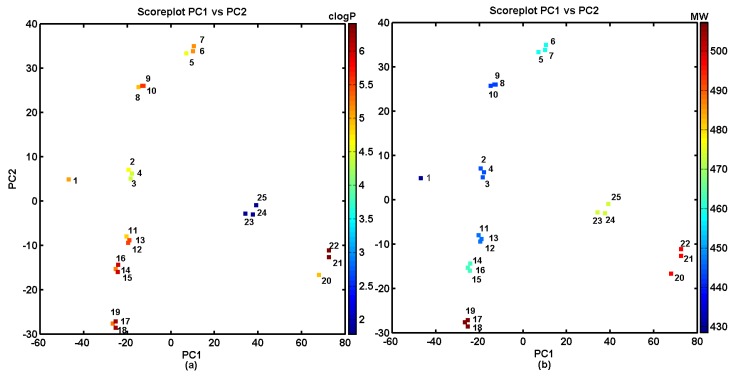
Projection of carbamates **1**–**25** on plane defined by the first versus second principal components for Dragon descriptors with calculated lipophilicity clogP (**a**) and molecular weights MW (**b**). Colors indicate the numerical values of clogP and MW.

The detailed investigation of the most active BChE objects **2**–**4**, projected on the PC1 versus PC2 plain, and color-coded according to calculated lipophilicity (Figure 2a) and molecular weight (Figure 2b), indicated that lower lipophilicity (clogP < 5) was accompanied by lower molecular weight (MW < 450 Da). Not surprisingly, the lipophilic profile for compounds is directly related to their structural composition (chemotypes) and is partially mirrored by the MW values as well.

**Figure 3:**
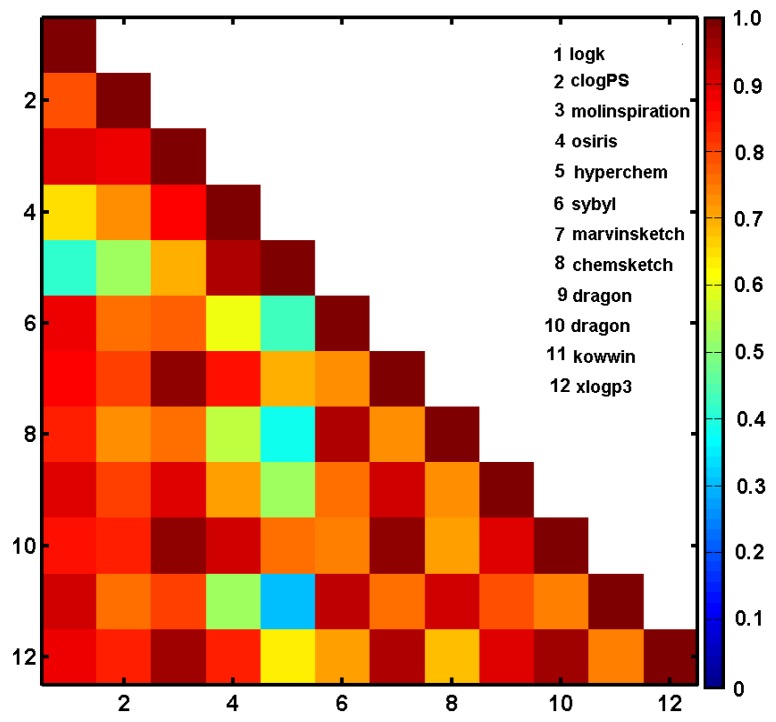
Matrix of correlation coefficients of linear relationships between experimental lipophilicity (log*k*) and lipophilicity calculated using different programs.

In silico estimation and experimental specification of the lipophilic values for the ensemble of silicon-based carbamates was carried out using a few logP predictors; e.g., clogPS, Molinspirations, OSIRIS, HyperChem 7.0, Sybyl X, MarvinSketch 15, ACD/ChemSketch 2015, Dragon6.0, Kowwin and XlogP3. The numerical values of the theoretically-estimated partition coefficients and the empirically-specified log*k* parameters are reported in Table S11 in the Supplementary Materials. The corresponding logP estimators deduced by the set of alternative programs were (inter-)correlated with each other and cross-compared with the experimental values, as shown in Figure 3 and Table S12.

Basically, a relatively high correlation of the estimated values of logP with the experimental log*k* was recorded (r > 0.85) for the investigated set of compounds with the exception of HyperChem 7.0 and OSIRIS clogP predictor. As a matter of fact, some observed variations in clogP values are probably the consequence of various computational principles implemented in the software; e.g., descriptor, atom or fragment-based and/or training databases used at the modelling stage (models are as good as the training data applied) [58]. Moreover, we employed the iterative variable elimination procedure (IVE-PLS) on the overall clogP matrix (X_25×11_) to select clogPS, Molinspirations, OSIRIS, HyperChem 7.0, Sybyl X and Dragon6.0 software as significant contributors to the final model in, namely, consensus clogP specification. The averaged values of the indicated clogP estimators were correlated with log*k* data with the correlation coefficient r = 0.82, because not only the best inter-correlated logP estimators were indicated.

**Figure 4:**
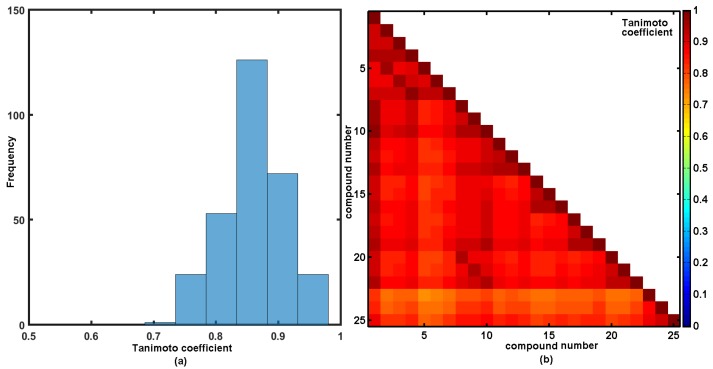
Distribution of Tanimoto coefficients (**a**) and the triangular matrix of Tanimoto coefficients (**b**) for silicon-based carbamates **1**–**25**.

The idea of specifying a numerical measure of the intermolecular similarity based on the degree of the resemblance between two objects, each described by a number of attributes, has been widely applied in chemical information systems. Chemical structures are frequently represented by descriptors encoding molecular structure/property in a numerical format [59]. The descriptor-based similarity comparison is used as a quantitative assessment of the pair-wise structural relatedness, since it is difficult to evaluate molecular similarity ‘by the eye’. In fact, various types of the similarity measures have been described so far [60]. Hamming or Euclidean measures are basically used for the ‘relative’ distance comparisons, whereas the Tanimoto coefficient is preferred as a similarity measure between a pair of the independent molecules in, namely, the ‘absolute’ comparison. In this study, the similarity between two molecules was quantified using Tanimoto coefficient evaluated between pairs of the OpenBabel fingerprint [61]. The distribution of Tanimoto coefficients for the investigated series of compounds is illustrated in Figure 4a, with the highest frequency recorded in range of 0.84 < T < 0.89, respectively. Given a set of 25 molecules, a symmetrical matrix of T_25×25_ was calculated showing the structural dissimilarities of nitro-substituted isomers (compounds **23**–**25**) from the remaining ones, as illustrated in Figure 4b.

**Figure 5:**
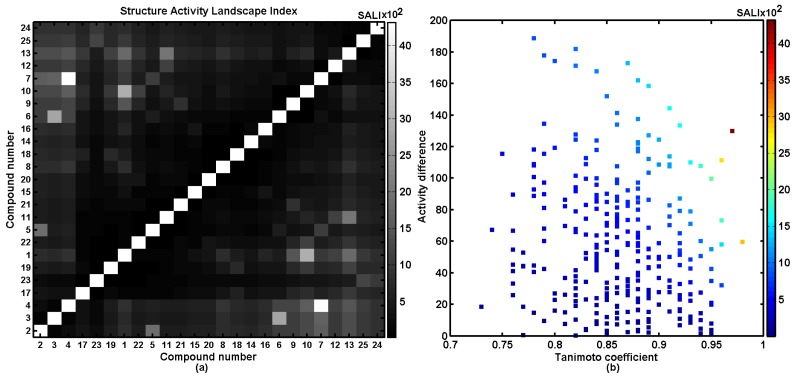
The gray-scaled structure–activity landscape indexes (SALI) plot with compounds ordered by increasing BChE activity (**a**), and a neighboring plot (**b**) for silicon-based carbamates **1**–**25**.

The integration of the pairwise comparison of the chemical structure and biological response profile provides a consistent visualization framework for the elucidation of SAR trends; e.g., continuity regions and/or activity cliffs [10,11,12,13,14]. A graphical representation of chemical similarity versus biological activity can be derived by a systematic profiling of structure–activity landscape indexes (SALI). The identification of activity cliffs depends critically on the accessibility of chemically-related compounds with noticeably large activity variations; therefore, BChE values were taken into consideration in the SALI calculation. Obviously, for closely related molecules (e.g., stereoisomers where T→1), SALI→infinity; thus, such values were replaced with the next largest SALI value. The symmetrical heatmap representation of SALI in the grayscale for the scrutinized series of compounds is presented in Figure 5a, with axes that correspond to molecules sorted in increasing BChE inhibitory activity and a legend indicating the range of SALI values. With the molecules ordered by BChE activity, two types of spots can be immediately noted. The white ones represent pairs of compounds exhibiting the highest SALI values, whereas the black ones indicate pairs revealing the minimum SALI values. The lighter block located in the right lower part of the map indicates pairs of molecules that potentially form the BChE activity cliff, where potent molecules **2**–**4** are accompanied by the inactive *meta*/*para* substituted compounds **6**/**7** (-OCH_3_), **9**/**10** (-CH_3_), **12**/**13** (-F) and **24**/**25** (-NO_2_), respectively. Moreover, the unfavorable structural modifications can be detected on the neighborhood plot, where the similar pairs of compounds (T > 0.85) are plotted versus variations in the biological activity (Δ_BChE_ > 100) and color-coded by higher SALI values, as illustrated in Figure 5b. The hints provided, based on the graphical/numerical SALI representations, prompt further dense sampling of the indicated SAR-variations with the potential compound synthesis as well as the activity specification.

### 2.5. Probability-Oriented Pharmacophore Mapping

**Figure 6:**
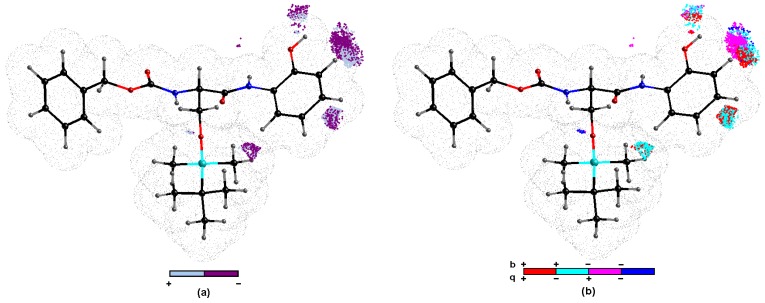
Spatial sectors with the largest contribution into BChE inhibitory potency selected by CoMSA IVE-PLS for **17**/**8** training/test set samplings. Color coded contributions (**a**) and four possible combinations of mean charge q and correlation coefficient b values (**b**) for silicon-based carbamates **1**–**25**. Reference compound **2** is illustrated as the most potent molecule.

The systematic probing of the crucial functional motifs in order to possibly specify a set of essential 3D steric/electronic/lipophilic features, namely pharmacophore, is common for the majority of SAR studies. Basically, the specific congeneric series of compounds that shares the same structural core (chemotype scaffold) is scrutinized. In fact, QSAR modelers frequently aspire to predict the activities of new molecules; however, the predictive ability of model is directly dependent on the training/test division. In real-world drug discovery, a model with ‘good retrospective performance does not necessarily exhibit good prospective performance’ (Kubinyi paradox) [62]. Restricting ourselves to single numerical measure of $q_{cv}^{2}$/$q^{2}{}_{test}$ can be misleading; therefore, we proposed ‘consensus-based’ pharmacophore generation using stochastic model validation (SMV) methodology [9,24,44]. Firstly, the original dataset of 25 compounds was recurrently sampled into **17**/**8** training/test subgroups (ratio 2 to 1). Basically, it is usually problematic to arbitrarily specify the training/test ratio, especially in case where the external validation should be carried out a number of times [63]. Luckily, it was technically feasible to investigate the whole pool of systematically generated training/test populations ($C_{25}^{8}$ ≈ 10^6^) for CoMSA BChE modeling. Obviously, the predictive performance of the test subset was related to the preferential selection of objects into the training set. The frequency distribution of compounds in the test group for models with acceptable $q_{cv}^{2}$ ≥ 0.5 (better than flipping a coin) revealed that mostly inactive *para* isomers (**5**, **7**, **10**, **13** and **16**) were indicated. Subsequently, the regions of fairly high model ability and predictability were sampled to produce an ‘average’ pharmacophore pattern according to the procedure described elsewhere [64]. The pharmacophore pattern illustrated in Figure 6 was produced using the pre-selected cut-off = 0.5 value, and further filtering of 50% of the CoMSA descriptors with a negligible statistical importance for the BChE inhibitory profile was applied. Colors code the impact of the descriptors on the BChE inhibitory potency to indicate the areas of positive and negative potency contribution (Figure 6a) and the four combinations of charge values versus the mean regression coefficients (Figure 6b), respectively. The potentially negative impact on the BChE inhibitory potency, mainly due to steric and/or electronic factors, is graphically represented by the dark 3D polyhedrals, while the bright ones depict the spatial areas that were foreseen to be occupied by atom/substituent in order to improve the molecular inhibitory profile, as shown in Figure 6a. It appears that bulky substitution at *ortho* position of phenyl group can increment the BChE inhibitory potency, as illustrated by white areas next to the hydroxyl group of the most potent compound **2**. On the other hand, the continuous increase in bulk at *ortho* region does not necessarily lead to further potency growth that is in line with the observed inhibitory response reported in Table 1 (e.g., -OH versus -OCH_3_). Noticeably, the increase in the bulkiness at *meta* substitution seems to be unfavorable structural modification, as illustrated by the dominant dark spheres in the close proximity of this position in Figure 6a. The above observation confirms the tendency recorded for the positional isomers of the examined carbamates where BChE IC_50_ can be generally ranked as *meta < ortho*. Similarly, substituents attached directly to phenyl ring at *para* position contribute unfavorably to the BChE inhibition activity, as suggested by the dark spheres in Figure 6a and data reported in Table 1. A similar tendency was observed for the frequency distribution in the test set for the best models, as mentioned earlier; not only is the bulk of substituents an important factor, but also the distribution of the electron density. One can conclude that the presence of the positively as well as negatively charged groups at the *meta* position is unfavorable to the BChE inhibition, as illustrated by magenta and cyan color in Figure 6b. The spatially-allowed areas at *ortho* position should be occupied by positively charged atoms/groups, as depicted by red color in Figure 6b; however, the surface/volume enhancement can be detrimental to the inhibition profile of the investigated compounds.

To some extent, the ‘consensus’ 3D-QSAR approach provides a graphical representation of an ‘average’ pharmacophore pattern, with some hints indicating the functional groups that can potentially increase/decrease the inhibitory potency of the silicon-based carbamates.

### 2.6. Molecular Docking Study of BChE and PET Activities

In order to derive more detailed insight into the guest–host interactions, 3D ligand-based approaches can be combined with the site-directed protein-based docking procedures, especially beneficial when a spatial geometry (or homology model) of the target responsible for the ADMET profile is accessible [65]. In the docking-driven SAR modeling, the enthalpically and/or enthropically favorable ligand-targeting factors can be deduced directly from the protein (or enzyme) binding site and the ligand property/descriptor–coded structural space, respectively. How to accurately relate the ligand-target interactions to pharmacological or toxicological effects is still questionable; however, the utility of the intuitive docking procedures for generating guest-host poses in the structure-based drug-design (SBDD) is widely accepted as a complimentary approach to the traditional ligand-guided procedures (LBDD) [66]. To compare SBDD and LBDD findings for the AChE/BChE crystallographic data with commercially-marketed drug molecules (e.g., GLT or RIV) is urgently needed.

**Figure 7:**
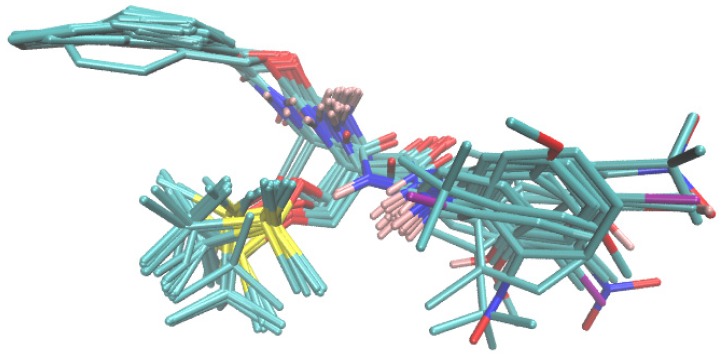
Docking-driven alignment of BChE silicon-based inhibitors in the enzyme active site AC2.

The crystallographic geometry of BChE with a catalytic core (CC) determined at a resolution of 2.6 Å in the liganded state (holo) with the RIV analog 3-[(1*R*)-1-(dimethylamino)ethyl]-4-hydroxyphenyl ethyl(methyl)carbamate was downloaded from the Protein Data Bank repository (PDB entry code: 6EUL) [51,67]. The AutoDock Vina program was used to (re)dock the RIV analogue in the active site (AC2) of the enzymatic chain A that is comprised of six amino acids: Asn68, Ile69, Asp70, Trp82, Thr120 and Pro285, along with a 1,2-ethanediol molecule (EDO605). The implementation of a new scoring function within AutoDock Vina improved, considerably, the speed and accuracy of docking algorithm compared to other programs that were tested extensively [68]. Obviously, the ligand-enzyme interactions are dependent on the pose (conformation and the relative orientation) of both the host and guest molecule; therefore, the entire anti-BChE population was scrutinized for rough guidelines to be compared with the drug–enzyme interaction pattern, although the silicon-based carbamates are structurally distinct from the reference RIV analogue. In fact, an automated docking procedure confirmed our intuitive scaffold-driven alignment selection, as illustrated in Figure 7.

**Figure 8:**
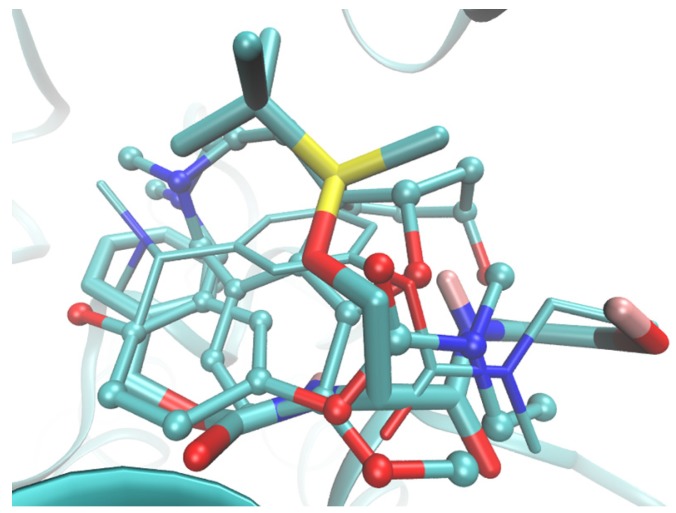
Active site of BChE enzyme (depicted as ribbons) bounded with GLT (displayed as balls and sticks), RIV (illustrated as thin sticks) and the most potent molecule 2 (presented as thick sticks).

GLT, as the most potent BChE inhibitor, was docked in the active site of the BChE as well. Despite structural differences between RIV, GLT and **1**–**25** carbamate series, some similarities in the atom spatial distribution can be observed for negatively charged atoms (nitrogen and oxygen), as shown in Figure 8.

**Figure 9:**
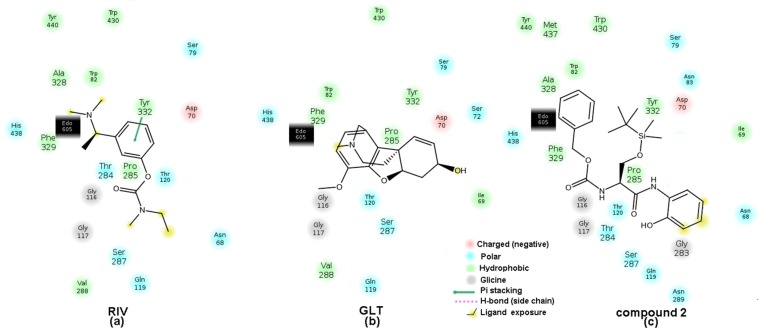
2D-based comparison of binding pattern for RIV (**a**), GLT (**b**) and the most active BChE inhibitor, **2**, produced by the Maestro program.

**Figure 10:**
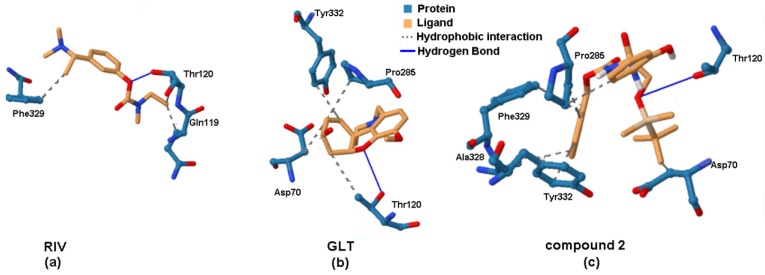
3D-based comparison of the binding pattern for RIV (**a**), GLT (**b**) and the most active BChE inhibitor **2**, produced by PLIP program.

The visualization of the host–guest interacting modes was conducted using planar (2D) and spatial (3D) maps generated by Schrödinger Maestro software and the Protein–Ligand Interaction Profiler (PLIP) [69], revealing, generally, two types of non-bonding interactions: hydrogen-bond and hydrophobic ones. The two and three-dimensional binding patterns for the marketed drug molecules (GLT and RIV) and the most potent anti-BChE molecule **2** are displayed in Figure 9 and Figure 10, respectively.

The hydroxyl group of Thr120 seems to play a vital role as a hydrogen donor when forming the hydrogen bond (HB) with the ether (Figure 10a,b) and carbonyl oxygen of molecule **2**, as shown in Figure 10c. Moreover, both terminal phenyl rings were specified as crucial building sub-blocks for the hydrophobic interactions with Pro285 or Tyr332 that partially correspond to our previous ligand-oriented findings. Regarding all interactions foreseen for the entire set of the silicon-based **1**–**25** carbamates, the hydrophobic interactions were dominated by the following order of amino acid residues: Tyr332, Ala328, Pro285, Phe329, Trp82 and Asp70, whereas Thr120 was generally indicated as the hydrogen bond donor (HBD) to the negatively charged oxygen of the carbamate carbonyl or etheric group. Interestingly, for the most potent BChE carbamates **3** and **4**, the Thr120-based hydrogen bond is additionally accompanied by a hydrogen bond generated with Asn289. Unfortunately, there was no clear explanation of the differences in the anti-BChE profile for *ortho/meta/para*-positioned carbamates provided by the docking study.

**Figure 11:**
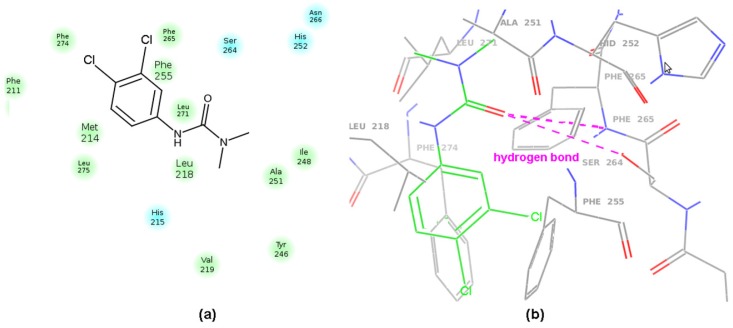
2D-based (**a**) and 3D-based (**b**) interacting modes of DCMU in the photosystem II (PSII) binding pocket produced by Maestro software.

Due to the lack of the X-ray data on PSII-herbicide complexes, the experimental orientation and conformation of herbicides targeting the Q_B_ binding pocket still remain unknown [70]. In the theoretical studies of herbicide-resistant mutants, different interaction modes have been suggested for various families of herbicides [71]. In consequence, the molecular docking study can provide valuable knowledge about the mechanism of herbicide binding indicating the specific amino acids in the Q_B_ site as well. The initial atomic coordinates of the D1 protein were retrieved from the monomeric X-ray structure of cyanobacterial Photosystem II at 2.9 Å resolution (PDB entry code: 3BZ1). The docking pocket was set to a 20 Å cube centered at the Q_B_ ring in EC9 binding site the central part of which is formed with hydrophobic Phe and Leu residues; e.g., Phe(211, 255, 265, 274) and Leu(218, 271), respectively. Carbamate and amide-like herbicides are regarded as PET inhibitors in PSII that cause displacement of Q_B_ from the binding site of the D1 protein [72,73,74]. Hence, the molecular docking for the most potent DCMU herbicide resulted in it interacting with the OH group of D1-Ser264 and the backbone NH group of D1-Phe265 with the DCMU carbonyl group (see Figure 11a,b). That is in line with the previously published findings of DCMU resistance by Ser264 mutations [42].

**Figure 12:**
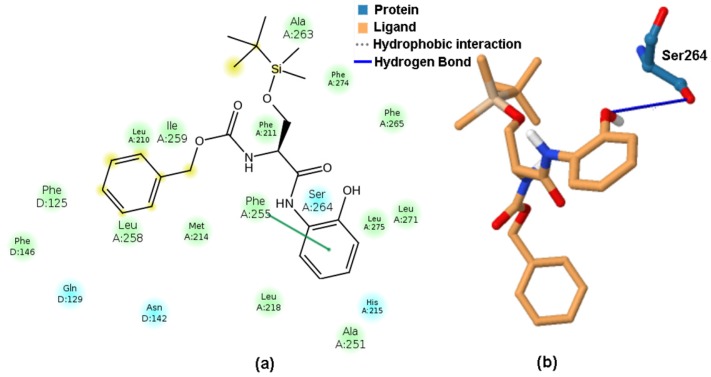
2D-based and 3D-based interacting modes for the most potent PET inhibitor (molecule **2**) produced by Maestro (**a**) and PLIP (**b**) programs.

Moreover, the docking studies were conducted for the whole set of silicon-based **1**–**25** carbamates to examine the host–guest interacting mode, indicating basically hydrophobic interactions, with Phe275 and Phe265 as the dominant type of non-binding interactions. Interestingly, only the most potent PET inhibitor (molecule **2**) was predicted to be hydrogen-bonded with D1-Ser264, as illustrated in planar (Figure 12a) and spatial (Figure 12b) interacting modes, respectively.

Obviously, we are profoundly aware that both ligand and structure-driven approaches have their own limitations; therefore, a consensus methodology i.e., the combination of the pharmacophore mapping with target-tailored procedures was proposed in the investigation of the multifaceted inhibitor-enzyme interactions [75].

### 2.7. In Vitro Viability Assay

The preliminary in vitro screening of the most effective compounds and their influence on cell viability was performed using the human monocytic leukemia THP-1 cell line, as described previously [23]. The effect on viability was evaluated as the IC_50_ value (compound concentration causing 50% inhibition of cell population proliferation) (see Table 1). A compound is considered as cytotoxic when it demonstrates a toxic effect on cells at the concentration up to 10 μM [76], and the highest tested concentration that was used for the toxicity assay was three times this value.

Treatment with the selected compounds led to decrease in viability of the cells only in case of compounds **2**, **3** and **4**; nevertheless, for comparison, the IC_50_ of camptothecin was 0.16 ± 0.07 µM [77]. The IC_50_ values of these compounds are shown in Table 1, and ranged from 3 to ca. 6.5 µM. The antiproliferative effect is probably related to substitution of anilide ring by hydroxy moiety, and this effect decreased as follows: *ortho* > *meta* > > > *para*. Surprisingly, the nitro derivatives **23** and **25** showed a non-significant antiproliferative effect, as described recently [78]. No lethal effect was shown by compounds **6**, **7** and **25**, the potent AChE inhibitors. Additionally compound **1**, a potent inhibitor of PET, did not lead to a significant lethal effect on THP-1 cells. Based on these observations, it can be concluded that these compounds can be considered non-toxic agents for the subsequent design of novel drugs.

## 3. Materials and Methods

### 3.1. General Methods

All reagents were purchased from Sigma-Aldrich and Acros, respectively. Anhydrous dichloromethane was distilled from calcium hydride. TLC experiments were performed on alumina-backed silica gel 60 F254 plates (Merck, Darmstadt, Germany). The plates were illuminated under UV (254 nm). The melting points were determined on Böetius PHMK 05 (Franz Küstner Nachf). Infrared (IR) spectra were collected on a Smart MIRacle^TM^ ATR ZnSe for the Nicolet^TM^ Impact 410 FT-IR Spectrometer (Thermo Scientific). All ^1^H, ^19^F and ^13^C spectra were specified using a JEOL ECZR 400 MHz NMR spectrometer (400 MHz for ^1^H, 101 MHz for ^13^C and 376 MHz for ^19^F, JEOL) in CDCl_3_. Chemical shifts (δ) are reported in ppm. High-resolution mass spectra were measured on LTQ Orbitrap XL^TM^Hybrid Ion Trap-Orbitrap Fourier Transform Mass Spectrometer (Thermo Scientific) with injection into HESI II in the positive mode.

### 3.2. Chemistry

#### 3.2.1. General Procedure Used to Synthesize the Carbamates **1**–**24**

(*2S*)-2-{[(Benzyloxy)carbonyl]amino}-3-hydroxypropanoic acid (10.01 g, 41.84 mM), *tert*-butyldimethylsilyl chloride (9.50 g, 63.31 mM) and imidazole (11.42 g, 167.75 mM) were dissolved in dry DMF (400 mL). The reaction mixture was heated to 60 °C under inert gas atmosphere for 72 h. The reaction was monitored by thin-layer chromatography (ethyl acetate: methanol/9:1). The solvent was evaporated in vacuo and petroleum ether was added to the rest (400 mL), and subsequently extracted with 5% NaHCO_3_ (3 × 140 mL). Aqueous layers were acidified to pH 3 with 1M KHSO_4_ and extracted with EtOAc (3 × 150 mL). Combined organic layers were washed with brine (200 mL) and dried with MgSO_4_. The solvent was evaporated under reduced pressure and the residue was triturated with EtOAc/petroleum ether to give white crystals of *(2S)*-2-{[(benzyloxy)carbonyl]amino}-3-[(*tert*-butyldimethylsilyl)oxy]propanoic acid. Yield 87% [79]; Mp 80–82 °C; ^1^H NMR (CDCl_3_) δ: 7.31–7.40 (m, 5 H), 5.62 (d, *J* = 8.23 Hz, 1 H), 5.14 (d, *J* = 5.03 Hz, 2 H), 4.46 (d, *J* = 7.78 Hz, 1 H), 4.13 (dd, *J* = 10.06, 2.29 Hz, 1 H), 3.86 (dd, *J* = 10.06, 3.20 Hz, 1 H), 0.87 (s, 9 H), 0.05 (s, 6 H); ^13^C NMR (CDCl_3_) δ: 175.0, 156.1, 136.1, 128.5, 128.2, 128.1, 67.2, 63.3, 55.6, 25.7, 18.2, −5.6; IR (cm^−1^): 3651w, 3437w, 3241w, 2992 w, 2782w, 1706s, 1567m, 1467w, 1378w, 1282w, 1229m, 1090s, 894s, 781m; HR-MS: for C_17_H_28_NO_5_Si [M + H]^+^ calculated 354.1731 *m/z*, found 354.1733 *m/z*.

*(2S)*-2-{[(Benzyloxy)carbonyl]amino}-3-[(*tert*-butyldimethylsilyl)oxy]propanoic acid (353.50 mg, 1.00 mM) and dry TEA (202.00 mg, 2.00 mM) were dissolved in dry THF (8 mL) and cooled down to −5 °C. Ethyl chloroformate (108.52 mg, 1.00 mmol) was added dropwise to this solution during 15 min. The reaction mixture was stirred for 1 h at 0 °C, and then a specific derivative of aniline (1.00 mM) was added. The reaction mixture was monitored by thin-layer chromatography and reaction times were from 7 to 24 h according to used aniline. When the reaction was finished, water (10 mL) was added and the solution was extracted with EtOAc (3 × 15 mL). Combined organic layers were washed with 1M HCl (12 mL), saturated NaHCO_3_ (12 mL) and brine (10 mL), and dried with anhydrous Na_2_SO_4_. The solvent was evaporated in vacuo and the residue was purified using column chromatography to give compounds (**1**–**24**). For each product, the used mobile phases are presented in Table 2.

*Benzyl-N-[(1S)-2-[(tert-butyldimethylsilyl)oxy]-1-(phenylcarbamoyl)ethyl]carbamate* (**1**): Yield 83%; Mp 93–96 °C; ^1^H NMR (CDCl_3_) δ: 8.47 (br s, 1 H), 7.50 (d, *J* = 7.89 Hz, 2 H), 7.30–7.42 (m, 7 H), 7.13 (t, *J* = 7.43 Hz, 1 H), 5.82 (br s, 1 H), 5.17 (s, 2 H), 4.35 (br s, 1 H), 4.16 (d, *J* = 6.40 Hz, 1 H), 3.74 (dd, *J* = 9.43, 8.18 Hz, 1 H), 0.93 (s, 9 H), 0.13 (s, 6 H); ^13^C NMR (CDCl_3_) δ: 168.3, 156.1, 137.3, 136.0, 129.0, 128.6, 128.3, 128.1, 124.5, 119.8, 67.2, 63.2, 56.1, 25.8, 18.1, −5.5; IR (cm^−1^): 3170w, 2988w, 2885w, 1710w, 1678m, 1613s, 1571s, 1478m, 1421m, 1321m, 1150m, 1061m, 890s, 798s, 766m, 736m; HR-MS: for C_23_H_32_N_2_O_4_Si [M + H]^+^ calculated 429.2204 *m/z*, found 429.2207 *m/z*.

*Benzyl N-[(1S)-2-[(tert-butyldimethylsilyl)oxy]-1-[(2-hydroxyphenyl)carbamoyl]ethyl]carbamate* (**2**): Yield 45%; Mp 115–117 °C; ^1^H NMR (CDCl_3_) δ: 8.71 (s, 1 H), 8.40 (s, 1 H), 7.34–7.41 (m, 5 H), 7.10–7.16 (m, 1 H), 6.99–7.06 (m, 2 H), 6.87 (t, *J* = 7.78 Hz, 1 H), 5.76 (br s, 1 H), 5.18 (s, 2 H), 4.45 (br s, 1 H), 4.18 (dd, *J* = 9.78, 3.14 Hz, 1 H), 3.79 (dd, *J* = 9.83, 6.86 Hz, 1 H), 0.90 (s, 9 H), 0.11 (s, 6 H); ^13^C NMR (CDCl_3_) δ: 170.0, 156.1, 148.6, 135.7, 128.7, 128.4, 128.2, 127.4, 124.9, 122.3, 120.4, 119.5, 67.6, 63.1, 56.3, 25.7, 18.2, −5.5; IR (cm^−1^): 3736w, 3440w, 3387w, 3362w, 2906w, 1742m, 1695s, 1628s, 1567s, 1510s, 1471m, 1421m, 1296s, 1222s, 1036m, 1004m, 944s, 883s, 801s, 795s; HR-MS: for C_23_H_32_N_2_O_5_Si [M + H]^+^ calculated 445.2153 *m/z*, found 445.2156 *m/z*.

*Benzyl N-[(1S)-2-[(tert-butyldimethylsilyl)oxy]-1-[(3-hydroxyphenyl)carbamoyl]ethyl]carbamate* (**3**): Yield 47%; oil; ^1^H NMR (CDCl_3_) δ: 8.50 (s, 1 H), 7.50 (br s, 1 H), 7.37 (br s, 6 H), 7.14 (t, *J* = 8.06 Hz, 1 H), 6.73 (d, *J* = 5.49 Hz, 1 H), 6.64 (dd, *J* = 8.06, 1.31 Hz, 1 H), 5.82 (br s, 1 H), 5.16 (s, 2 H), 4.40 (br s, 1 H), 4.10 (dd, *J* = 9.89, 3.60 Hz, 1 H), 3.78 (dd, *J* = 9.95, 7.09 Hz, 1 H), 0.88 (s, 9 H), 0.08 (s, 6 H); ^13^C NMR (CDCl_3_) δ: 168.9, 157.1, 156.3, 138.1, 135.8, 129.8, 128.6, 128.3, 128.2, 112.0, 111.2, 107.4, 67.5, 63.2, 56.7, 45.6, 25.7, 18.1, 9.9, −5.6; IR (cm^−1^): 3535m, 3361w, 3002w, 2907w, 2812w,1742w, 1684w, 1632m, 1579m, 1478w, 1414m, 1346m, 1166s, 1138s, 1067w, 889s, 798s, 752m; HR-MS: for C_23_H_32_N_2_O_5_Si [M + H]^+^ calculated 445.2153 *m/z*, found 445.2154 *m/z*.

*Benzyl N-[(1S)-2-[(tert-butyldimethylsilyl)oxy]-1-[(4-hydroxyphenyl)carbamoyl]ethyl]carbamate* (**4**): Yield 50%; Mp 66–69 °C; ^1^H NMR (CDCl_3_) δ: 8.28 (br s, 1 H), 7.37 (br s, 5 H), 7.21–7.29 (m, 2 H), 6.75 (d, *J* = 8.80 Hz, 2 H), 5.81 (br s, 1 H), 5.16 (s, 2 H), 4.33 (br s, 1 H), 4.08–4.17 (m, 1 H), 3.74 (dd, *J* = 9.55, 7.60 Hz, 1 H), 2.06 (s, 1 H), 0.9 (s, 9 H), 0.1 (s, 6 H); ^13^C NMR (CDCl_3_) δ: 168.4, 156.2, 153.4, 135.9, 129.6, 128.6, 128.3, 128.2, 122.3, 115.8, 67.4, 63.2, 56.2, 45.5, 25.8, 18.1, 10.0, −5.5; IR (cm^−1^): 3629m, 3355w, 3134w, 2992w, 2899w, 2824w, 1738w, 1667m, 1578s, 1482m, 1307m, 1140m, 1058w, 880s, 791m, 759m; HR-MS: for C_23_H_32_N_2_O_5_Si [M + H]^+^ calculated 445.2153 *m/z*, found 445.2154 *m/z*.

*Benzyl N-[(1S)-2-[(tert-butyldimethylsilyl)oxy]-1-[(2-methoxyphenyl)carbamoyl]ethyl]carbamate* (**5**): Yield 76%; Mp 50–53 °C; ^1^H NMR (CDCl_3_) δ: 8.70 (br s, 1 H), 8.37 (d, *J* = 8.0 Hz, 1 H), 7.39 (br s, 5 H), 7.03–7.10 (m, 1 H), 6.97 (t, *J* = 7.66 Hz, 1 H), 6.88 (d, *J* = 8.00 Hz, 1 H), 5.82 (br s, 1 H), 5.11–5.23 (m, 2 H), 4.37 (br s, 1 H), 4.14 (d, *J* = 7.09 Hz, 1 H), 3.84 (s, 3 H), 3.76 (t, *J* = 7.78 Hz, 1 H), 0.89 (s, 9 H), 0.10 (s, 6 H); ^13^C NMR (CDCl_3_) δ: 168.0, 156.5, 156.1, 136.0, 130.4, 128.6, 128.3, 128.2, 121.6, 114.1, 67.2, 63.2, 56.0, 55.4, 25.8, 18.1, −5.5; IR (cm^−1^): 3305w, 2971w, 2913w, 1711w, 1689w, 1619m, 1569m, 1476w, 1379w, 1312w, 1231m, 1091s, 889s, 792m, 707m; HR-MS: for C_24_H_34_N_2_O_5_Si [M + H]^+^ calculated 459.2310 *m/z*, found 459.2312 *m/z*.

*Benzyl N-[(1S)-2-[(tert-butyldimethylsilyl)oxy]-1-[(3-methoxyphenyl)carbamoyl]ethyl]carbamate* (**6**): Yield 61%; Mp 55–57 °C; ^1^H NMR (CDCl_3_) δ: 8.48 (br s, 1 H), 7.38 (br.s., 5 H), 7.19–7.28 (m, 2 H), 6.97 (d, *J* = 7.78 Hz, 1 H), 6.69 (dd, *J* = 8.23, 2.06 Hz, 1 H), 5.80 (br s, 1 H), 5.17 (s, 2 H), 4.33 (br s, 1 H), 4.15 (d, *J* = 5.60 Hz, 1 H), 3.80 (s, 3 H), 3.73 (t, *J* = 8.86 Hz, 1 H), 0.93 (s, 9 H), 0.13 (s, 6 H); ^13^C NMR (CDCl_3_) δ: 168.3, 160.1, 156.1, 138.5, 136.0, 129.7, 128.6, 128.3, 128.1, 112.0, 110.5, 105.4, 67.2, 63.2, 56.1, 55.2, 25.8, 18.1, −5.5; IR (cm^−1^): 3359w, 2909w, 2898w, 1713w, 1682w, 1631m, 1577m, 1503w, 1489w, 1332m, 1177mm 893s, 800m, 757w, 736w; HR-MS: for C_24_H_34_N_2_O_5_Si [M + H]^+^ calculated 459.2310 *m/z*, found 459.2311 *m/z*.

*Benzyl N-[(1S)-2-[(tert-butyldimethylsilyl)oxy]-1-[(4-methoxyphenyl)carbamoyl]ethyl]carbamate* (**7**): Yield 61%; Mp 118–125 °C; ^1^H NMR (CDCl_3_) δ: 8.32 (br s, 1 H), 7.32–7.44 (m, 7 H), 6.83–6.90 (m, 2 H), 5.81 (s, 1 H), 5.16 (s, 2 H), 4.33 (br s, 1 H), 4.15 (dd, *J* = 9.55, 3.37 Hz, 1 H), 3.80 (s, 3 H), 3.73 (dd, *J* = 9.55, 7.95 Hz, 1 H), 0.92 (s, 9 H), 0.11 (s, 6 H); ^13^C NMR (CDCl_3_) δ: 168.0, 156.4, 156.1, 135.9, 130.4, 128.9, 128.4, 128.1, 121.5, 114.2, 67.2, 63.2, 56.1, 55.4, 25.8, 18.1, −5.5; IR (cm^−1^): 3468w, 3002w, 2909w, 2781w, 1670m, 1581m, 1480m, 1379w, 1298m, 1276m, 1208m, 1095s, 893s, 788m, 753m, 707w; HR-MS: for C_24_H_34_N_2_O_5_Si [M + H]^+^ calculated 459.2310 *m/z*, found 459.2311 *m/z*.

*Benzyl N-[(1S)-2-[(tert-butyldimethylsilyl)oxy]-1-[(2-methylphenyl)carbamoyl]ethyl]carbamate* (**8**): Yield 54%; Mp 106–107 °C; ^1^H NMR (CDCl_3_) δ: 8.19 (br s, 1 H), 7.81 (d, *J* = 7.89 Hz, 1 H), 7.32–7.42 (m, 5 H), 7.16–7.25 (m, 2 H), 7.10 (t, *J* = 7.09 Hz, 1 H), 5.81 (s, 1 H), 5.12–5.23 (m, 2 H), 4.37 (br s, 1 H), 4.21 (dd, *J* = 9.49, 2.97 Hz, 1 H), 3.80 (dd, *J* = 9.83, 7.20 Hz, 1 H), 2.21 (br s, 3 H), 0.91 (s, 9 H), 0.13 (s, 6 H); ^13^C NMR (CDCl_3_) δ: 168.3, 156.2, 135.9, 135.2, 130.4, 129.1, 128.6, 128.3, 128.2, 126.7, 125.3, 122.9, 67.3, 63.2, 56.5, 25.8, 18.3, 17.7, −5.3, −5.5; IR (cm^−1^): 3348m, 2970m, 2925m, 2885m, 1710w, 1653w, 1453w, 1382w, 1328w, 1158w, 1090m, 1047s, 880m, 801w, 737w; HR-MS: for C_24_H_34_N_2_O_4_Si [M + H]^+^ calculated 443.2361 *m/z*, found 443.2366 *m/z*.

*Benzyl N-[(1S)-2-[(tert-butyldimethylsilyl)oxy]-1-[(3-methylphenyl)carbamoyl]ethyl]carbamate* (**9**): Yield 65%; Mp 92–93 °C; ^1^H NMR (CDCl_3_) δ: 8.39 (s, 1 H), 7.33–7.41 (m, 6 H), 7.25–7.30 (m, 1 H), 7.21 (t, *J* = 7.55 Hz, 1 H), 6.95 (d, *J* = 7.32 Hz, 1 H), 5.79 (br s, 1 H), 5.17 (dd, *J* = 12.24, 1.94 Hz, 2 H), 4.33 (br s, 1 H), 4.15 (dd, *J* = 9.49, 3.20 Hz, 1 H), 3.74 (dd, *J* = 8.00, 1.49 Hz, 1 H), 2.35 (s, 3 H), 0.93 (s, 9 H), 0.12 (br s, 6 H); ^13^C NMR (CDCl_3_) δ: 168.2, 156.1, 138.9, 137.2, 136.0, 128.8, 128.6, 128.3, 128.1, 125.3, 120.6, 117.0, 67.2, 63.2, 56.2, 25.8, 21.5, 18.1, −5.5; IR (cm^−1^): 3319m, 2970m, 2888w, 1710w, 1674m, 1599m, 1456m 1325m, 1190m, 1154m, 1090m, 1047s, 880s, 801m, 755m, 727m; HR-MS: for C_24_H_34_N_2_O_4_Si [M + H]^+^ calculated 443.2361 *m/z*, found 443.2365 *m/z*.

*Benzyl N-[(1S)-2-[(tert-butyldimethylsilyl)oxy]-1-[(4-methylphenyl)carbamoyl]ethyl]carbamate* (**10**): Yield 72%; Mp 111–112 °C; ^1^H NMR (CDCl_3_) δ: 8.38 (br s, 1 H), 7.37 (d, *J* = 6.40 Hz, 7 H), 7.13 (d, *J* = 8.12 Hz, 2 H), 5.81 (br s, 1 H), 5.17 (s, 2 H), 4.33 (br s, 1 H), 4.15 (d, *J* = 6.52 Hz, 1 H), 3.73 (t, *J* = 8.75 Hz, 1 H), 2.33 (s, 3 H), 0.92 (s, 9 H), 0.12 (s, 6 H); ^13^C NMR (CDCl_3_) δ: 168.1, 156.1, 136.0, 134.8, 134.1, 129.5, 128.6, 128.3, 128.1, 119.9, 67.2, 63.2, 56.1, 25.8, 20.8, 18.1, −5.5; IR (cm^−1^): 3309m, 2970m, 2881m, 1710w, 1681w, 1592w, 1446m, 1382m, 1328w, 1086m, 1044s, 880m, 734w; HR-MS: for C_24_H_34_N_2_O_4_Si [M + H]^+^ calculated 443.2361 *m/z*, found 443.2366 *m/z*.

*Benzyl N-[(1S)-2-[(tert-butyldimethylsilyl)oxy]-1-[(2-fluorophenyl)carbamoyl]ethyl]carbamate* (**11**): Yield 47%; Mp 70–72 °C; ^1^H NMR (CDCl_3_) δ: 8.74 (br s, 1 H), 8.31 (t, *J* = 7.72 Hz, 1 H), 7.31–7.45 (m, 5 H), 7.03–7.19 (m, 3 H), 5.79 (br s, 1 H), 5.12–5.24 (m, 2 H), 4.38 (br s, 1 H), 4.17 (d, *J* = 6.75 Hz, 1 H), 3.75 (dd, *J* = 9.55, 8.06 Hz, 1 H), 0.91 (s, 9 H), 0.13 (s, 6 H); ^13^C NMR (CDCl_3_) δ: 168.5, 156.1, 152.5 (d, ^1^*J*_CF_ = 243.7 Hz, 1C), 136.0, 128.6, 128.3, 128.2, 125.9 (d, ^3^*J*_CF_ = 10.6 Hz, 2C), 124.6, 124.5 (d, ^4^*J*_CF_ = 2.9 Hz, 1C), 121.7, 114.9 (d, ^2^*J*_CF_ = 19.3 Hz, 2C), 67.3, 63.3, 56.4, 25.8, 18.2, −5.6; IR (cm^−1^): 3544w, 3312w, 3163w, 2970w, 2881w, 1720m, 1678m, 1628s, 1574s, 1510m, 1414m, 1332m, 1286s, 1143s, 1061m, 1022m, 896s, 801s, 716m; HR-MS: for C_23_H_31_FN_2_O_4_Si [M + H]^+^ calculated 447.2110 *m/z*, found 447.2112 *m/z*.

*Benzyl N-[(1S)-2-[(tert-butyldimethylsilyl)oxy]-1-[(3-fluorophenyl)carbamoyl]ethyl]carbamate* (**12**): Yield 73%; Mp 84–88 °C; ^1^H NMR (CDCl_3_) δ: 8.54 (br s, 1 H), 7.47 (d, *J* = 10.75 Hz, 1 H), 7.32–7.42 (m, 5 H), 7.22–7.31 (m, 1 H), 7.10 (d, *J* = 7.89 Hz, 1 H), 6.82 (td, *J* = 8.20, 2.23 Hz, 1 H), 5.77 (br s, 1 H), 5.17 (s, 2 H), 4.34 (br s, 1 H), 4.15 (dd, *J* = 9.43, 3.26 Hz, 1 H), 3.74 (dd, *J* = 9.66, 7.83 Hz, 1 H), 0.92 (s, 9 H), 0.12 (s, 6 H); ^13^C NMR (CDCl_3_) δ: 168.5, 163.0 (d, ^1^*J*_CF_ = 244.7 Hz, 1C), 156.2, 138.8 (d, ^3^*J*_CF_ = 10.6 Hz, 1C), 135.9, 130.1 (d, ^3^*J*_CF_ = 8.7 Hz, 1C), 128.6, 128.3, 128.2, 115.0, 111.2 (d, ^2^*J*_CF_ = 22.2 Hz, 2C), 107.5, 107.2, 67.3, 63.2, 56.3, 25.8, 18.1, −5.5; IR (cm^−1^): 3512w, 3351w, 2977w, 2913w, 1713m, 1685m, 1628m, 1567m, 1460w, 1346w, 1167m, 1090m, 1054m, 887s, 798s, 734m; HR-MS: for C_23_H_31_FN_2_O_4_Si [M + H]^+^ calculated 447.2110 *m/z*, found 447.2112 *m/z*.

*Benzyl N-[(1S)-2-[(tert-butyldimethylsilyl)oxy]-1-[(4-fluorophenyl)carbamoyl]ethyl]carbamate* (**13**): Yield 68%; Mp 110–112 °C; ^1^H NMR (CDCl_3_) δ: 8.43 (br s, 1 H), 7.45 (dd, *J* = 8.35, 4.69 Hz, 2 H), 7.31–7.41 (m, 5 H), 7.02 (t, *J* = 8.63 Hz, 2 H), 5.77 (br s, 1 H), 5.16 (s, 2 H), 4.33 (br s, 1 H), 4.15 (dd, *J* = 9.43, 3.14 Hz, 1 H), 3.74 (dd, *J* = 9.49, 7.89 Hz, 1 H), 0.92 (s, 9 H), 0.11 (s, 6 H); ^13^C NMR (CDCl_3_) δ: 168.3, 159.4 (d, ^1^*J*_CF_ = 244.7 Hz, 1C), 156.2, 135.9, 133.4, 128.6, 128.3, 128.2, 121.6 (d, ^3^*J*_CF_ = 7.7 Hz, 2C), 115.7 (d, ^2^*J*_CF_ = 23.1 Hz, 2C), 67.3, 63.2, 56.2, 25.8, 18.1, −5.5; IR (cm^−1^): 3366w, 3184w, 2981w, 2896w, 1738w, 1685m, 1631m, 1588s, 1539m, 1482m, 1428m, 1375w, 1324m, 1289m, 1210m, 1175m, 1143m, 940m, 873s, 801s, 748m; HR-MS: for C_23_H_31_FN_2_O_4_Si [M + H]^+^ calculated 447.2110 *m/z*, found 447.2112 *m/z*.

*Benzyl N-[(1S)-2-[(tert-butyldimethylsilyl)oxy]-1-[(2-chlorophenyl)carbamoyl]ethyl]carbamate* (**14**): Yield 68%; oil; ^1^H NMR (CDCl_3_) δ: 8.75 (br s, 1 H), 8.35 (d, *J* = 8.12 Hz, 1 H), 7.23–7.40 (m, 7 H), 7.05 (td, *J* = 7.75, 1.32 Hz, 1 H), 5.77 (s, 1 H), 5.17 (q, *J* = 12.20 Hz, 2 H), 4.39 (br s, 1 H), 4.19 (d, *J* = 7.66 Hz, 1 H), 3.80 (dd, *J* = 9.89, 6.46 Hz, 1 H), 0.87 (s, 9 H), 0.09 (d, *J* = 7.66 Hz, 6 H); ^13^C NMR (CDCl_3_) δ: 168.6, 156.1, 135.9, 134.1, 129.0, 128.6, 128.3, 128.2, 127.6, 124.9, 123.1, 121.6, 67.4, 63.3, 57.1, 25.8, 18.3, −5.4, −5.5; IR (cm^−1^): 3530w, 3341w, 2970w, 2881w, 2835w, 1756w, 1678m, 1621s, 1571s, 1482m, 1457m, 1410m, 1332m, 1286w, 1161m, 1093w, 1061w, 904s, 801s, 712w; HR-MS: for C_23_H_31_ClN_2_O_4_Si [M + H]^+^ calculated 463.1814 *m/z*, found 463.1819 *m/z*.

*Benzyl N-[(1S)-2-[(tert-butyldimethylsilyl)oxy]-1-[(3-chlorophenyl)carbamoyl]ethyl]carbamate* (**15**): Yield 83%; Mp 80–81 °C; ^1^H NMR (CDCl_3_) δ: 8.48 (br s, 1 H), 7.60 (s, 1 H), 7.17–7.38 (m, 7 H), 7.07 (d, *J* = 7.78 Hz, 1 H), 5.73 (br s, 1 H), 5.13 (s, 2 H), 4.30 (br s, 1 H), 4.11 (dd, *J* = 9.38, 2.97 Hz, 1 H), 3.71 (dd, *J* = 9.55, 7.83 Hz, 1 H), 0.89 (s, 9 H), 0.08 (s, 6 H); ^13^C NMR (CDCl_3_) δ: 168.5, 156.2, 138.4, 135.9, 134.7, 130.0, 128.6, 128.4, 128.2, 124.5, 120.0, 117.7, 67.4, 63.1, 56.3, 25.8, 18.1, −5.5; IR (cm^−1^): 3355m, 2970m, 2903w, 2693w, 1727w, 1674m, 1610s, 1560m, 1499m, 1435w, 1321m, 1289m, 1176m, 1143m, 1097m, 1054s, 899s, 801s, 748m; HR-MS: for C_23_H_31_ClN_2_O_4_Si [M + H]^+^ calculated 463.1814 *m/z*, found 463.1818 *m/z*.

*Benzyl N-[(1S)-2-[(tert-butyldimethylsilyl)oxy]-1-[(4-chlorophenyl)carbamoyl]ethyl]carbamate* (**16**): Yield 92%; Mp 101–104 °C; ^1^H NMR (CDCl_3_) δ: 8.47 (s, 1 H), 7.42 (d, *J* = 8.69 Hz, 2 H), 7.31–7.38 (m, 5 H), 7.24–7.29 (m, 2 H), 5.76 (br s, 1 H), 5.14 (s, 2 H), 4.32 (s, 1 H), 4.12 (dd, *J* = 9.83, 3.43 Hz, 1 H), 3.72 (dd, *J* = 9.61, 7.78 Hz, 1 H), 0.89 (s, 9 H), 0.09 (s, 6 H); ^13^C NMR (CDCl_3_) δ: 168.4, 156.2, 135.9, 129.4, 129.0, 128.9, 128.6, 128.3, 128.2, 121.0, 67.3, 63.12, 56.3, 25.8, 18.1, −5.5; IR (cm^−1^): 3366w, 3216w, 3152w, 3091w, 2974w, 2878w, 1177w, 1628m, 1571s, 1503m, 1432m, 1325s, 1275m, 1179m, 1154s, 1061s, 890s, 798m, 759m, 716w; HR-MS: for C_23_H_31_ClN_2_O_4_Si [M + H]^+^ calculated 463.1814 *m/z*, found 463.1823 *m/z*.

*Benzyl N-[(1S)-2-[(tert-butyldimethylsilyl)oxy]-1-[(2-bromophenyl)carbamoyl]ethyl]carbamate* (**17**): Yield 15%; oil; ^1^H NMR (CDCl_3_) δ: 8.69 (br s, 1 H), 8.34 (d, *J* = 8.23 Hz, 1 H), 7.53 (dd, *J* = 8.00, 1.14 Hz, 1 H), 7.31–7.43 (m, 6 H), 6.96–7.04 (m, 1 H), 5.77 (s, 1 H), 5.12–5.27 (m, 2 H), 4.40 (br s, 1 H), 4.19–4.25 (m, 1 H), 3.83 (dd, *J* = 9.95, 5.72 Hz, 1 H), 0.87 (s, 9 H), 0.09 (s, 6 H); ^13^C NMR (CDCl_3_) δ: 168.6, 156.1, 135.9, 135.2, 132.2, 128.6, 128.4, 128.3, 125.4, 121.8, 113.7, 67.4, 63.2, 57.3, 25.8, 18.3, −5.4, −5.5; IR (cm^−1^): 3512w, 3355w, 2970w, 2888w, 1770w, 1702m, 1621m, 1560m, 1489w, 1403w, 1328w, 1143s, 1090s, 1054s, 912s, 798s, 734m; HR-MS: for C_23_H_31_BrN_2_O_4_Si [M + H]^+^ calculated 507.1309 *m/z*, found 507.1318 *m/z*.

*Benzyl N-[(1S)-2-[(tert-butyldimethylsilyl)oxy]-1-[(3-bromophenyl)carbamoyl]ethyl]carbamate* (**18**): Yield 63%; Mp 79–80 °C; ^1^H NMR (CDCl_3_) δ: 8.52 (br s, 1 H), 7.77 (s, 1 H), 7.38 (br s, 6 H), 7.23–7.28 (m, 1 H), 7.15–7.21 (m, 1 H), 5.77 (br s, 1 H), 5.17 (s, 2 H), 4.34 (br s, 1 H), 4.14 (dd, *J* = 9.61, 3.20 Hz, 1 H), 3.74 (dd, *J* = 9.66, 7.72 Hz, 1 H), 0.92 (s, 9 H), 0.12 (s, 6 H); ^13^C NMR (CDCl_3_) δ: 168.5, 156.2, 138.6, 135.9, 130.3, 128.6, 128.4, 128.2, 127.5, 122.8, 122.6, 118.2, 67.3, 63.1, 56.2, 25.8, 18.1, −5.5; IR (cm^−1^): 3376m, 2974m, 2899w, 1717m, 1678m 1631m, 1560m, 1507m, 1442w, 1328w, 1286m, 1178m, 1097m, 1054m, 911s, 801m, 744m; HR-MS: for C_23_H_31_BrN_2_O_4_Si [M + H]^+^ calculated 507.1309 *m/z*, found 507.1315 *m/z*.

*Benzyl N-[(1S)-2-[(tert-butyldimethylsilyl)oxy]-1-[(4-bromophenyl)carbamoyl]ethyl]carbamate* (**19**): Yield 61%; Mp 117–120 °C; ^1^H NMR (CDCl_3_) δ: 8.49 (br s, 1 H), 7.32–7.46 (m, 9 H), 5.77 (br s, 1 H), 5.16 (s, 2 H), 4.33 (br s, 1 H), 4.14 (dd, *J* = 9.49, 3.20 Hz, 1 H), 3.73 (dd, *J* = 9.66, 7.72 Hz, 1 H), 0.91 (s, 9 H), 0.11 (s, 6 H); ^13^C NMR (CDCl_3_) δ: 168.4, 156.1, 136.4, 135.9, 132.0, 128.6, 128.3, 128.2, 121.4, 117.1, 67.3, 63.1, 56.3, 25.8, 18.1, −5.5; IR (cm^−1^): 3351w, 3213w, 3145w, 3088w, 2981w, 2885w, 1717w, 1701w, 1628m, 1571s, 1503m, 1418m, 1325m, 1268m, 1173m, 1165m, 1090m, 1040s, 887s, 798m, 759w; HR-MS: for C_23_H_31_BrN_2_O_4_Si [M + H]^+^ calculated 507.1309 *m/z*, found 507.1319 *m/z*.

*Benzyl N-[(1S)-2-[(tert-butyldimethylsilyl)oxy]-1-{[2-(trifluoromethyl)phenyl]carbamoyl}ethyl]carbamate* (**20**): Yield 15%; Mp 54–56 °C; ^1^H NMR (CDCl_3_) δ: 8.65 (br s, 1 H), 8.22 (d, *J* = 8.12 Hz, 1 H), 7.53–7.65 (m, 2 H), 7.39 (br s, 5 H), 7.22–7.29 (m, 1 H), 5.75 (s, 1 H), 5.18 (d, *J* = 4.92 Hz, 2 H), 4.39 (s, 1 H), 4.23 (d, *J* = 8.81 Hz, 1 H), 3.82 (dd, *J* = 10.01, 5.77 Hz, 1 H), 0.87 (s, 9 H), 0.09 (d, *J* = 8.69 Hz, 6 H); ^13^C NMR (CDCl_3_) δ: 168.9, 156.1, 135.9, 134.7, 132.9, 128.6, 128.4, 128.3, 126.1 (q, ^3^*J*_CF_ = 4.8 Hz, 2C), 124.7, 124.2, 123.9 (q, ^1^*J*_CF_ = 272.6 Hz, 1C), 120.4 (q, ^2^*J*_CF_ = 29.9 Hz, 1C), 67.5, 63.1, 57.1, 25.7, 18.3, −5.6; IR (cm^−1^): 3341w, 3173w, 2981w, 2903w, 2824w, 1749w, 1701w, 1617s, 1578s, 1471m, 1410m, 1343m, 1304m, 1180m, 1147s, 1054m, 883s, 798s, 716m; HR-MS: for C_24_H_31_F_3_N_2_O_4_Si [M + H]^+^ calculated 497.2078 *m/z*, found 497.2078 *m/z*.

*Benzyl N-[(1S)-2-[(tert-butyldimethylsilyl)oxy]-1-{[3-(trifluoromethyl)phenyl]carbamoyl}ethyl]carbamate* (**21**): Yield 52%; Mp 58–61 °C; ^1^H NMR (CDCl_3_) δ: 8.66 (br s, 1 H), 7.79 (s, 1 H), 7.70 (d, *J* = 7.78 Hz, 1 H), 7.45 (t, *J* = 7.78 Hz, 1 H), 7.39 (br s, 6 H), 5.77 (br s, 1 H), 5.17 (s, 2 H), 4.37 (br s, 1 H), 4.16 (dd, *J* = 9.38, 3.43 Hz, 1 H), 3.76 (dd, *J* = 9.61, 7.78 Hz, 1 H), 0.92 (s, 9 H), 0.12 (s, 6 H); ^13^C NMR (CDCl_3_) δ: 168.7, 156.2, 137.9, 135.9, 131.4 (q, ^2^*J*_CF_ = 32.8 Hz, 1C), 129.6, 128.6, 128.4, 128.2, 123.75 (q, ^1^*J*_CF_ = 272.6 Hz, 1C), 122.8, 121.0, 116.6 (q, ^3^*J*_CF_ = 3.9 Hz, 2C), 67.4, 63.1, 56.3, 25.7, 18.1, −5.5; IR (cm^−1^): 3177w, 2995w, 2899w, 1727w, 1684w, 1631s, 1597s, 1489s, 1389s, 1286s, 1190s, 1150s, 1036m, 955w, 869m, 816m, 759m, 723m; HR-MS: for C_24_H_31_F_3_N_2_O_4_Si [M + H]^+^ calculated 497.2078 *m/z*, found 497.2082 *m/z*.

*Benzyl N-[(1S)-2-[(tert-butyldimethylsilyl)oxy]-1-{[4-(trifluoromethyl)phenyl]carbamoyl}ethyl]carbamate* (**22**): Yield 55%; Mp 125–127 °C; ^1^H NMR (CDCl_3_) δ: 8.69 (br s, 1 H), 7.55–7.64 (m, 4 H), 7.38 (br s, 5 H), 5.78 (br s, 1 H), 5.17 (s, 2 H), 4.37 (br s, 1 H), 4.12–4.19 (dd, *J* = 9.38, 2.86 Hz, 1 H), 3.76 (dd, *J* = 9.66, 7.83 Hz, 1 H), 0.92 (s, 9 H), 0.12 (s, 6 H); ^13^C NMR (CDCl_3_) δ: 168.8, 156.2, 139.5, 135.8, 128.6, 128.4, 128.2, 126.2 (q, ^2^*J*_CF_ = 32.8 Hz, 1C), 126.3 (q, ^3^*J*_CF_ = 3.9 Hz, 2C), 123.9 (q, ^1^*J*_CF_ = 272.6 Hz, 1C), 119.4, 67.4, 63.1, 56.3, 25.8, 18.1, −5.5; IR (cm^−1^): 3355w, 3213w, 3148w, 3099w, 2977w, 2874w, 1749w, 1628m, 1578m, 1482m, 1428m, 1350s, 1286m, 1190s, 1143s, 1076m, 1040m, 887s, 798m, 759m; HR-MS: for C_24_H_31_F_3_N_2_O_4_Si [M + H]^+^ calculated 497.2078 *m/z*, found 497.2078 *m/z*.

*Benzyl N-[(1S)-2-[(tert-butyldimethylsilyl)oxy]-1-[(2-nitrophenyl)carbamoyl]ethyl]carbamate* (**23**): Yield 23%; oil; ^1^H NMR (CDCl_3_) δ: 8.81 (d, *J* = 8.35 Hz, 1 H), 8.24 (d, *J* = 8.35 Hz, 1 H), 7.31–7.47 (m, 7 H), 7.19–7.25 (m, 1 H), 5.82 (d, 1 H), 5.21 (s, 2 H), 4.43 (s, 1 H), 4.26 (d, 1 H), 3.85 (dd, *J* = 10.18, 4.46 Hz, 1 H), 0.82 (s, 9 H), 0.04 (d, *J* = 15.32 Hz, 6 H); ^13^C NMR (CDCl_3_) δ: 169.0, 156.3, 143.7, 143.0, 135.8, 128.6, 128.5, 128.3, 125.1, 119.2, 67.5, 63.0, 56.5, 25.8, 18.1, −5.5; IR (cm^−1^): 3462w, 3387w, 3230w, 2967m, 2903w, 1735w, 1628s, 1539s, 1446m, 1368s, 1300m, 1236w, 1172m, 1047m, 876s, 798m, 759m, 709m; HR-MS: for C_23_H_31_N_3_O_6_Si [M + H]^+^ calculated 474.2055 *m/z*, found 474.2056 *m/z*.

*Benzyl N-[(1S)-2-[(tert-butyldimethylsilyl)oxy]-1-[(3-nitrophenyl)carbamoyl]ethyl]carbamate* (**24**): Yield 53%; Mp 84–86 °C; ^1^H NMR (CDCl_3_) δ: 8.83 (br s, 1 H), 8.38 (s, 1 H), 7.97 (dd, *J* = 8.23, 1.37 Hz, 1 H), 7.88 (d, *J* = 7.89 Hz, 1 H), 7.49 (t, *J* = 8.18 Hz, 1 H), 7.38 (br s, 5 H), 5.78 (br s, 1 H), 5.18 (s, 2 H), 4.40 (br s, 1 H), 4.17 (dd, *J* = 9.66, 3.26 Hz, 1 H), 3.78 (dd, *J* = 9.83, 7.43 Hz, 1 H), 0.92 (s, 9 H), 0.12 (s, 6 H); ^13^C NMR (CDCl_3_) δ: 168.9, 156.3, 148.5, 138.5, 135.8, 129.9, 128.6, 128.4, 128.2, 125.3, 119.0, 114.6, 67.5, 63.0, 56.4, 25.7, 18.1, −5.5; IR (cm^−1^): 3351w, 3230w, 2970w, 2871w, 1681w, 1621m, 1553s, 1450s, 1414s, 1375s, 1321m, 1279m, 1136m, 1086m, 1040s, 955w, 879s, 794s, 744m, 715m; HR-MS: for C_23_H_31_N_3_O_6_Si [M + H]^+^ calculated 474.2055 *m/z*, found 474.2059 *m/z*.

#### 3.2.2. General Procedure Used for the Synthesis of Carbamate **25**

*(2S)*-2-{[(Benzyloxy)carbonyl]amino}-3-[(*tert*-butyldimethylsilyl)oxy]propanoic acid (300.00 mg, 0.85 mM) and 4-nitroaniline (176.01 mg, 1.28 mM) were dissolved in dry pyridine (5 mL). The mixture was cooled down to −15 °C and phosphorous oxychloride (169.43 mg, 1.11 mM) was added dropwise. The reaction mixture was stirred under inert atmosphere at this temperature for 2 h and at room temperature overnight. The reaction was quenched with ice water (25 mL) and extracted with EtOAc (3×20 mL). The combined organic layers were washed with saturated NaHCO_3_ (15 mL), brine (15 mL), dried with MgSO_4_ and concentrated in vacuo. The residue was purified by column chromatography (EtOAc/petroleum ether (1:3)) to afford yellow crystals of benzyl *N*-[(*1S*)-2-[(*tert*-butyldimethylsilyl)oxy]-1-[(4-nitrophenyl)carbamoyl]ethyl]carbamate (**25**).

Benzyl N-[(1S)-2-[(tert-butyldimethylsilyl)oxy]-1-[(4-nitrophenyl)carbamoyl]ethyl]carbamate [79] (**25**): Yield 51%; Mp 111–115 °C; ^1^H NMR (CDCl_3_) δ: 8.86 (s, 1 H), 8.19–8.24 (m, 2 H), 7.66 (d, *J* = 8.69 Hz, 2 H), 7.33–7.42 (m, 5 H), 5.73 (s, 1 H), 5.18 (t, *J* = 12.35 Hz, 2 H), 4.37 (br s, 1 H), 4.18 (dd, *J* = 10.06, 3.66 Hz, 1 H), 3.77 (dd, *J* = 9.83, 7.55 Hz, 1 H), 0.92 (s, 9 H), 0.13 (s, 6 H); ^13^C NMR (CDCl_3_) δ: 169.0, 156.3, 143.7, 143.0, 135.8, 128.6, 128.5, 125.1, 119.2, 67.5, 63.0, 56.5, 25.8, 18.1, −5.5; IR (cm^−1^): 3391w, 3230w, 2977w, 2871w, 2831w, 2831w, 1695m, 1635s, 1578m, 1514m, 1453s, 1375s, 1282s, 1204m, 1133s, 1079s, 1044m, 880s, 798s, 759m; HR-MS: for C_23_H_31_N_3_O_6_Si [M + H]^+^ calculated 474.2055 *m/z*, found 474.2067 *m/z*.

### 3.3. Lipophilicity Measurement

The lipophilicity of synthesized compound was evaluated using Agilent 1200 Series HPLC instrument (Agilent technologies, Waldbron, Germany) controlled through the Chemstation software (version B.04.03). The separation was performed on ZORBAX Extend-C18 (3 × 150 mm, 3.5 µm) column (Agilent Technologies, Waldbron, Germany). Methanol (constituent A) and purified water (constituent B) were used as a mobile phase. Total flow rate was set up to 0.4 mL/min; the injection volume was 1 µL; and the column temperature was maintained at 25 °C. The detection wavelength of 210 nm was used. Retention factors of the compounds were measured under five different isocratic conditions in the 65:35–80:20 range (A:B; v/v), in duplicate. The log *k*_w_ values (indexes of lipophilicity) were estimated by linear regression analysis based on the following equation,

log *k = −S*Φ + log *k*_w_, (1)

where *logk* represents a logarithm of an individual isocratic retention factor, *Φ* is the organic phase concentration and *S* is a constant derived from linear regression analysis.

### 3.4. Evaluating In Vitro AChE and BChE-Inhibition Potencies

The ability of all the prepared compounds to inhibit AChE from electric eel (*Electrophorus electricus*) and BChE from equine serum (both purchased from Sigma, St. Louis, MO, USA) was determined in vitro using a modified Ellman’s method, as described in detail elsewhere [24,80,81,82,83]. The results are presented in Table 1.

### 3.5. Study of Inhibition of Photosynthetic Electron Transport (PET) in Spinach Chloroplasts

Chloroplasts were prepared from spinach (*Spinacia oleracea* L.) according to Kralova et al. [84]. The inhibition of photosynthetic electron transport (PET) in spinach chloroplasts was determined spectrophotometrically (Genesys 6, Thermo Scientific), using an artificial electron acceptor 2,6-dichlorophenol-indophenol (DCIPP) according to Kralova et al. [84], and the rate of photosynthetic electron transport was monitored as a photoreduction of DCPIP. The measurements were carried out in phosphate buffer (0.02 M, pH 7.2) containing sucrose (0.4 M), MgCl_2_ (0.005 M) and NaCl (0.015 M). The chlorophyll content in these experiments was 30 mg/L, and the samples were irradiated (~100 W/m^2^ with 10 cm distance) with a halogen lamp (250 W) using a 4 cm water filter to prevent warming of the samples (suspension temperature 22 °C). The compounds were dissolved in DMSO due to their limited water solubility. The applied DMSO concentration (up to 4%) did not affect the photochemical activity in spinach chloroplasts. The inhibitory efficiency of the compounds was expressed by IC_50_ values; i.e., by molar concentration of the compounds, causing a 50% decrease in the oxygen evolution rate relative to the untreated control. The comparable IC_50_ value for the selective herbicide 3-(3,4-dichlorophenyl)-1,1-dimethylurea, DCMU (Diuron^®^) was about 2.1 μM. The results are shown in Table 1.

### 3.6. In Vitro Viability Assay

Human monocytic leukemia THP-1 cells were used for in vitro antiproliferative assays. Cells were obtained from the European Collection of Cell Cultures (ECACC, Salisbury, UK) and routinely cultured in RPMI 1640 (Biosera, France) medium supplemented with 10% fetal bovine serum (Biosera, France), 2% l-glutamine, 1% penicillin and streptomycin (Biosera, France) at 37 °C with 5% CO_2_. Cells were passaged at approximately one-week intervals. The effect of the compounds on cell viability was determined using a Water Soluble Tetrazolium Salts-1 (WST-1, 2-(4-iodophenyl)-3-(4-nitrophenyl)-5-(2,4-disulfophenyl)-2*H*-tetrazolium) assay kit (Roche Diagnostics, Mannheim, Germany) according to the manufacturer’s instructions. The compounds were dissolved in DMSO and added in five increasing concentrations (0.37, 1.1, 3.3, 10 and 30 μM) to the cell suspension in the culture RPMI 1640 medium. The maximum concentration of DMSO (Sigma) in the assays never exceeded 0.1%. Subsequently, the cells were incubated at 37 °C with 5% CO_2_ for 24 h. For WST-1 assays, cells were seeded in triplicate into 96-well plates (5 × 10^4^ cells/well in 100 μL culture medium) using serum-free RPMI 1640 medium, and measurements were taken 24 h after the treatment with the compounds. The median inhibition concentration values, IC_50_, were deduced through the production of a dose-response curve. All data were evaluated using the Microsoft Excel. The results are shown in Table 1.

### 3.7. Building the Model and Molecular Modelling

Each molecular model was generated using the CACTVS/csed molecular editor. The initial spatial geometry of the molecules was specified using a CORINA 3D generator. The structural data conversion was conducted with the (inter)change file format converter OpenBabel. Sybyl-X 2.0/Certara software package running on a HP workstation with a Debian 6.0 operating system was used to perform the majority of the molecular modelling simulations. The standard Tripos force field (POWELL conjugate gradient algorithm) with a 0.01 kcal/mol energy gradient convergence criterion and a distant dependent dielectric constant were used to optimize the initial geometry of each compound (MAXMIN2 module). The partial atomic charges were specified with the Gasteiger–Hückel method implemented in Sybyl-X for the calculation of the electrostatic potential values. One 15-ordered atom trial alignment on the most active molecule **2** (Active Analogue Approach) was used to cover the whole bonding topology in the maximal common structure (MCS) using the atom FIT method, which is based on matching the positions of the atoms between the corresponding atom pairs.

The SONNIA software was employed in the CoMSA analysis to simulate 20 × 20 to 50 × 50 SOMs with a winning distance ranging from 0.2 to 2.0 [85]. SOM network was employed using the Cartesian coordinates of the molecular surfaces to generate a 2D map of the electrostatic potential for the superimposed molecules. Following the AAA approach, the most active molecule **2** was selected as a template molecule (reference compound). The output maps were reshaped into a 400 to 2500-element vector that was subsequently transformed by the PLS method implemented in the MATLAB programming environment.

The input crystallographic data of BChE and PSII inhibitor-enzyme complexes were taken from Protein Data Bank (PDB entry code: 6EUL and 1BZ1). Only EDO (1,2-ethanediol) and PL9 (2,3-dimethyl-5-(3,7,11,15,19,23,27,31,35-nonamethyl-2,6,10,14,18,22,26,30,34-hexatriacontanonaenyl-2,5-cyclohaxadiene-1,4-dione-2,3-dimethyl-5-solanesyl-1,4-benzoquinone) with the ionic cofactors (Fe^2+^ and Cl^−^) were retained in the active site AC2 of BChE and EC9 of PSII enzymes, respectively. The remaining heteroatoms, including water molecules, were erased from the input files prior to the calculations. The ligand/enzyme structural files were prepared in the pdbqt format using MGLTools’ inbuilt python scripts [61]. The ligand charges were calculated with semi-empirical PM3 method implemented in HyperChem software with default SCF settings. The docking pocket was set to a 20 Å cube with a 0.2 Å grid spacing centered on the RIV for BChE and PL9 for PSII aromatic rings, respectively. Different poses (default nine) were specified progressively on the basis of an energy-minimized ligand conformer using the AutoDock programme with the Lamarckian genetic algorithm and subsequently evaluated with the in-build united-atom scoring function [68]. Finally, planar (2D) and spatial (3D) visualizations of the predicted ligand-enzyme binding modes were produced using PyMol, Maestro, VMD and PLIP software [69].

The input crystallographic data of BChE and PSII inhibitor-enzyme complexes were taken from Protein Data Bank (PDB entry code: 6EUL and 1BZ1). Only EDO (1,2-ethanediol) and PL9 (2,3-dimethyl-5-(3,7,11,15,19,23,27,31,35-nonamethyl-2,6,10,14,18,22,26,30,34-hexatriacontanonaenyl-2,5-cyclohaxadiene-1,4-dione-2,3-dimethyl-5-solanesyl-1,4-benzoquinone) with the ionic cofactors (Fe^2+^ and Cl^−^) were retained in the active site AC2 of BChE and EC9 of PSII enzymes, respectively. The remaining heteroatoms, including water molecules, were erased from the input files prior to the calculations. The ligand/enzyme structural files were prepared in the pdbqt format using MGLTools’ inbuilt python scripts [61]. The ligand charges were calculated with semi-empirical PM3 method implemented in HyperChem software with default SCF settings. The docking pocket was set to a 20 Å cube with a 0.2 Å grid spacing centered on the RIV for BChE and PL9 for PSII aromatic rings, respectively. Different poses (default nine) were specified progressively on the basis of an energy-minimized ligand conformer using the AutoDock programme with the Lamarckian genetic algorithm and subsequently evaluated with the in-build united-atom scoring function [68]. Finally, planar (2D) and spatial (3D) visualizations of the predicted ligand-enzyme binding modes were produced using PyMol, Maestro, VMD and PLIP software [69].

### 3.8. In Silico Lipophilicity Evaluation

A diverse set of freely or commercially accessible computer programs can be employed to specify the theoretical partition coefficient, for instance: 

AlogPS—an approach developed by Tetko et al. that is based on atom-type electrotopological-state (E-state) indices and neural networks;

milogP—a method provided by Molinspiration that enables practical logP calculations for almost all organic molecules as the sum of fragment-based contributions and correction factors;

ClogP—a fragment-based procedure implemented in Sybyl/Centara software that is able to accurately predict lipophilicity based on structure-dependent correction values taken from Hansch and Leo’s database;

HyperChem logP—an atom-additive method that estimates lipophilicity based on the individual atomic contributions following Ghose, Prichett and Crippen approach;

MarvinSketch logP—the overall lipophilicity of a molecule consists of the contributing values of its atoms, the types of which are redefined to accommodate electron delocalization and the contributions of ionic forms;

ChemSketch logP—a comprehensive, fragment-based algorithm methodology with high quality models specified on the basis of empirical data. Well-characterized logP contributions were compiled for atoms, structural fragments and intramolecular interactions derived from >12000 experimental logP values;

Dragon AlogP—the statistical estimators of the Ghose-Crippen-Viswanadhan model were calculated based on the known experimental logP on the training set of 8364 compounds. The overall estimation of the lipophilic atomic-based constant is provided with the contribution of 115 atom types;

Dragon MlogP—the theoretical partition coefficient includes VdW volume and Moriguchi polar parameters as correction factors. MlogP model is composed of a regression equation that is based on 13 structural parameters which were assessed by a trainings group of 1230 organic molecules;

Kowwin—evaluates the log octanol-water partition coefficient of chemicals applying the atom/fragment contribution method;

XlogP3—an atom-additive method with well-defined correction factors, that employs an optimized atom-typing approach calibrated on a large training set.

### 3.9. Structure Activity Landscape Index

The systematic profiling of potency-similarity relationships in the form of the activity landscape provides a sharp picture of favorable structural modifications that are necessary to identify the activity cliffs [11]. The smoothness of this landscape can be numerically quantified using the structure–activity landscape index (SALI) introduced as follows:

(2) $$SALI_{x,y}=\frac{\left|A_{x}-A_{y}\right|}{1-sim(x,y)},$$

where $A_{x}$ and $A_{y}$ are activities of the *x*-th and *y*-th molecules and *sim*(*x*,*y*) is the similarity coefficient between corresponding molecules [14]. In other words, SALI assigns the score that combines the pairwise similarity and the variations in the activity, respectively. It was shown that the overall appearance of the symmetric SALI matrix minimally depends on the specific similarity metric; therefore, the Tanimoto coefficient was employed in the fingerprint space similarity analysis [7]. Despite many obstacles, the Tanimoto metric shed light on the number of features that two molecules have in common to the total number of unique features present in each molecule according to the following formula:

(3) $$T(x,y)=\frac{n_{xy}}{\left(n_{x}+n_{y}-n_{xy}\right)},$$

where $n_{xy}$ is the number of bits set into 1 shared in the fingerprint of the molecule x i y; $n_{x}$ is the number of bits set into 1 in the molecule x; and $n_{y}$ is the number of bits set into 1 in the molecule y. The ratio of T(x,y) > 0.8 indicates a criterion of a high structural/property similarity to the target structure; however, the validity of this assumption is still questionable since similar compounds do not necessarily interact with the same target in the same manner. SALI values should be adequately sampled for structures that represent similar chemotypes but different potency profiles, to identify activity cliffs.

### 3.10. Principal Component Analysis and Partial Least Squares with Iterative Variable Elimination

Principal Component Analysis (PCA) is a linear projection method that condenses a multidimensional data (organized in matrix X_m×n_ with the rows corresponding to m objects and the columns corresponding to n parameters) into a few explanatory principal components (PCs) [86]. PCA is a classic method of data exploration, which allows one to reduce data dimensionality, because a restricted ensemble of the orthogonal PCs creates a basis of the lower-dimensional space. In the method, input matrix X is decomposed into three matrices called scores (T), loadings (P) and the residual matrix (E), respectively. In other words, the PCA model with f principal components for a data matrix X can be presented according the following equation:

X = TP^T^+ E, (4)

where *X* is a data matrix.

Provided that the reduction of data dimensionality is effective, it is possible to use the score vectors and loading vectors to visualize and to interpret the relationships between the objects and the parameters in the input matrix. The similarities between the original data objects are evaluated using the distance metrics.

Partial least squares (PLS) is a multivariate statistical method that can analyze the input data with strong co-linearity and model, simultaneously. A relationship between response data (dependent variable) Y and an ensemble of descriptors (independent variables) X is expressed briefly in the form of the following formula:

Y = X *b* + e, (5)

where *b* is a vector of the regression coefficients and *e* is a vector of errors.

Structural information encoded by a myriad of descriptors shows a significant degree of overlap; therefore, over-parametrization occurs frequently in QSAR studies. In other words, numerous descriptors show restrained variability and/or are strongly (inter)correlated as well. To avoid random noise, the recurrent variable selection method conjugated with the PLS procedure (IVE-PLS) was effectively employed in the mD-QSAR studies. In fact, it can be regarded as an extension of the single-step uninformative variable elimination algorithm (UVE) that was originally proposed by Centner et al. for the selection of the variables to be eradicated [87]. Vaguely, the whole computer implementation was comprised of four basic step including: (**i**) standard PLS analysis with LOO-CV to evaluate the performance of the PLS model, (**ii**) elimination of a matrix column with the lowest abs(mean(b)/std(b)) value, (**iii**) standard PLS analysis of the new matrix without the column eliminated in stage (**ii**) and (**iv**) recurrent repetition of steps (**i**)–(**iii**) to maximize the LOO parameter.

## 4. Conclusions

(1) A set of novel 25 silicon-based carbamate derivatives as potential acetyl and butyrylcholinesterase inhibitors was synthesized and were characterized by their in vitro inhibition profiles and the selectivity indexes. Moreover, the compounds were sampled for their inhibition potential against photosynthetic electron transport in spinach (*Spinacia oleracea L.*) chloroplasts. In fact, some of the newly prepared molecules revealed comparable or even better inhibitory activities compared to marketed drugs (rivastigmine or galanthamine) and commercially applied pesticides (Diuron^®^), respectively. Generally, more compounds exhibited better inhibition potency towards AChE; however, a wider activity span (maximum value less minimum ones) was observed for BChE. Notably, benzyl *N*-[(*1S*)-2-[(*tert*-butyldimethylsilyl)oxy]-1-[(2-hydroxyphenyl)carbamoyl]- ethyl]carbamate (**2**) and benzyl *N*-[(*1S*)-2-[(*tert*-butyldimethylsilyl)oxy]-1-[(3-hydroxyphenyl)- carbamoyl]ethyl]carbamate (**3**) are characterized by fairly high selective indexes (SIs). Specifically, compound **2** has the lowest IC_50_ value that corresponds quite well with galanthamine inhibition activity, while the inhibitory profiles of molecules **3** and benzyl *N*-[(*1S*)-2-[(*tert*-butyldimethylsilyl)oxy]-1-[(4-hydroxyphenyl)carbamoyl]ethyl]carbamate (**4**) are in line with rivastigmine activity. 

(2) The SAR-driven similarity evaluation of physicochemical properties for the carbamates examined was reported to indicate the activity cliffs using similarity-activity landscape index for BChE inhibitory response values. The similarity-mediated evaluation of property profiles was conducted using the PCA procedure on the pool of Dragon descriptors. The corresponding logP estimators deduced by the set of alternative programs were (inter-)correlated with each other and cross-compared with the experimental log*k* values, respectively. In this study, the similarity between two molecules was quantified using Tanimoto coefficient evaluated between pairs of the OpenBabel fingerprint. A graphical representation of chemical similarity versus biological activity was derived from the systematic profiling of structure–activity landscape indexes (SALI). The identification of activity cliffs depends, critically, on the accessibility of chemically related compounds with noticeably large activity variations; therefore, BChE values were taken into consideration in the SALI calculation. The hints provided based on the graphical/numerical SALI representations prompt further dense sampling of the indicated SAR-variations with the potential compound syntheses as well as the activity specifications.

(3) The ‘indirect’ ligand-based and ‘direct’ protein-mediated in silico approaches were applied to specify electronic/steric/lipophilic factors that were potentially valid for (Q)SAR modeling of the carbamate analogues. The stochastic model validation was used to generate an ‘average’ 3D-QSAR pharmacophore pattern. It seems that bulky substitution of phenyl group at *ortho* position can increment the BChE inhibitory potency. On the other hand, the continuous increase in bulk at *ortho* region does not necessarily lead to further potency growth that is in line with the observed inhibitory response (e.g., -OH versus -OCH_3_). Noticeably, the increase in the bulkiness at *meta* substitution seems to be unfavorable for structural modification. The above observation confirms the tendency recorded for the positional isomers of the examined carbamates where BChE IC_50_ can be generally ranked as *meta* < *ortho*. 

(4) The target-oriented molecular docking was employed to (re)arrange the spatial distribution of the ligand property space for BChE enzyme and PSII system as well. Despite structural differences between RIV, GLT and **1**–**25** carbamate series, some similarities in the atom spatial distribution can be observed for negatively charged atoms of nitrogen and oxygen, respectively. The OH group of Thr120 seems to play a vital role as hydrogen donor in forming the hydrogen bond (HB) with the ether and carbonyl oxygen of the most potent molecule **2**. Moreover, both terminal phenyl rings were specified as crucial building (sub)blocks for the hydrophobic interactions with Pro285 or Tyr332, which partially corresponds to our previous ligand-oriented findings. The molecular docking for the most potent DCMU herbicide resulted in interaction with the OH group of D1-Ser264 and the backbone NH group of D1-Phe265 with the DCMU carbonyl group. Interestingly, only the most potent PET inhibitor (molecule **2**) was predicted to be hydrogen-bonded with D1-Ser264.

Despite the ligand and structurally-driven approach’s limitations, a consensus methodology combining the pharmacophore mapping and the target-tailored procedures was proposed for investigating of the multifaceted inhibitor–enzyme interactions.

## Figures and Tables

**Table 1:**
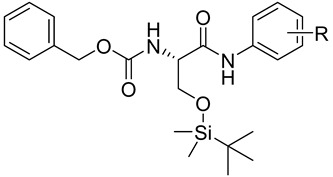
Structures of the carbamate derivatives **1**–**25**, experimental log *k*_w_ values and electronic Hammett’s σ parameters; in vitro PET, AChE and BChE inhibition (IC_50_ [μM]) compared with standards 3-(3,4-dichlorophenyl)-1,1-dimethylurea (DCMU) rivastigmine (RIV) and galanthamine (GLT) and in vitro viability (WST) assays (IC_50_ [μM]) of investigated compounds. ChE inhibition is expressed as the mean ± SD (*n* = 3 experiments).

No.	R	log *k*_w_	σ *^a^*	PET IC_50_ [µM]	AChE IC_50_ [µM]	BChE IC_50_ [µM]	SI *^b^*	WST IC_50_ [µM]
**1.**	H	6.52 ± 0.13	0	**4.90**	53.52 ± 0.40	73.29 ± 0.28	0.73	>30
**2.**	2-OH	6.00 ± 0.04	−0.38	**0.48**	42.15 ± 2.06	**8.37 ± 0.03**	**5.04**	3.08 ± 0.07
**3.**	3-OH	5.59 ± 0.03	0.12	**4.94**	59.70 ± 0.43	**19.08 ± 0.17**	**3.13**	3.47 ± 0.08
**4.**	4-OH	5.40 ± 0.03	−0.37	483	36.22 ± 1.65	**22.70 ± 0.24**	**1.60**	6.43 ± 0.29
**5.**	2-OCH_3_	5.85 ± 0.21	−0.28	>500	38.63 ± 1.81	81.47 ± 0.21	0.47	–
**6.**	3-OCH_3_	6.26 ± 0.03	0.12	234	**29.39 ± 0.44**	130.30 ± 1.37	0.23	>30
**7.**	4-OCH_3_	6.09 ± 0.03	−0.27	>500	**28.37 ± 0.74**	152.54 ± 4.54	0.19	>30
**8.**	2-CH_3_	6.24 ± 0.02	−0.17	>500	46.41 ± 0.14	105.40 ± 2.78	0.44	–
**9.**	3-CH_3_	6.61 ± 0.03	−0.07	447	48.51 ± 3.77	131.22 ± 2.88	0.37	–
**10.**	4-CH_3_	6.62 ± 0.03	−0.17	452	45.04 ± 1.76	132.75 ± 2.46	0.34	–
**11.**	2-F	6.32 ± 0.02	0.06	51.5	43.03 ± 1.07	81.52 ± 4.81	0.53	>30
**12.**	3-F	6.44 ± 0.02	0.34	49.2	45.10 ± 1.43	160.29 ± 7.89	0.28	>30
**13.**	4-F	6.30 ± 0.02	0.06	22.9	62.65 ± 1.41	181.13 ± 10.05	0.35	>30
**14.**	2-Cl	6.74 ± 0.03	0.22	>500	47.15 ± 0.92	108.29 ± 1.74	0.44	–
**15.**	3-Cl	6.90 ± 0.02	0.37	332	48.12 ± 1.63	96.23 ± 5.29	0.50	–
**16.**	4-Cl	6.94 ± 0.03	0.23	>500	42.56 ± 0.23	115.98 ± 0.60	0.68	–
**17.**	2-Br	6.82 ± 0.03	0.22	354	46.27 ± 1.09	62.60 ± 5.59	0.74	–
**18.**	3-Br	7.05 ± 0.03	0.39	390	54.37 ± 0.83	107.33 ± 0.43	0.51	–
**19.**	4-Br	7.16 ± 0.04	0.23	42.3	53.18 ± 0.01	72.58 ± 3.72	0.73	>30
**20.**	2-CF_3_	6.90 ± 0.04	0.51	453	38.08 ± 0.16	104.17 ± 0.36	0.37	–
**21.**	3-CF_3_	7.32 ± 0.03	0.43	432	51.16 ± 1.03	88.87 ± 0.71	0.58	–
**22.**	4-CF_3_	7.53 ± 0.02	0.51	>500	42.79 ± 2.45	77.76 ± 0.97	0.55	–
**23.**	2-NO_2_	6.53 ± 0.06	0.77	43.2	46.14 ± 2.62	63.12 ± 1.14	0.73	>30
**24.**	3-NO_2_	6.40 ± 0.04	0.71	>500	49.42 ± 0.47	197.00 ± 11.28	0.25	–
**25.**	4-NO_2_	6.58 ± 0.04	0.78	253	**26.35 ± 0.84**	190.35 ± 6.61	0.14	>30
**DCMU**	–	–	–	2.1	–	–	–	–
**RIV**	–	–	–	–	50.10 ± 3.08	19.95 ± 0.31	1.46	–
**GLT**	–	–	–	–	4.0 ± 0.13	7.96 ± 0.59	0.56	–

*^a^* calculated using ACD/Percepta 14.0.0; *^b^* SI (index selectivity) = IC_50_ (AChE)/IC_50_ (BChE). A higher selectivity to BChE is indicated in bold.

**Table 2:** Mobile phases used for purification of the compounds **1–24**.

Comp.	Mobile phase
**1**	*n*-hexane/ethyl acetate (6:1)
**2**	2% TEA in 10% methanol in dichloromethane
**3**	2% TEA in 10% methanol in dichloromethane
**4**	2% TEA in 10% methanol in dichloromethane
**5**	*n*-hexane/ethyl acetate (4:1)
**6**	*n*-hexane/ethyl acetate (4:1)
**7**	*n*-hexane/ethyl acetate (4:1)
**8**	*n*-hexane/ethyl acetate (4:1)
**9**	*n*-hexane/ethyl acetate (6:1)
**10**	*n*-hexane/ethyl acetate (4:1)
**11**	1% DEA in *n*-hexane/ethyl acetate (6:1)
**12**	1% DEA in *n*-hexane/ethyl acetate (6:1)
**13**	1% DEA in *n*-hexane/ethyl acetate (6:1)
**14**	8% TEA in *n*-hexane/ethyl acetate (7:1)
**15**	*n*-hexane/ethyl acetate 9:1 → 6:1 → 4:1
**16**	1% DEA in *n*-hexane/ethyl acetate (6:1)
**17**	12% TEA in *n*-hexane/ethyl acetate (5:1)
**18**	1% DEA in *n*-hexane/ethyl acetate (6:1)
**19**	1% DEA in *n*-hexane/ethyl acetate (6:1)
**20**	1% DEA in *n*-hexane/ethyl acetate (6:1)
**21**	4% TEA in dichloromethane/*n*-hexane (5:1)
**22**	1% DEA in *n*-hexane/ethyl acetate (6:1)
**23**	1% DEA in *n*-hexane/ethyl acetate (2:1 → 3:1)
**24**	*n*-hexane/ethyl acetate (6:1 → 4:1)

## Supplementary Materials

Supplementary materials can be found at https://www.mdpi.com/1422-0067/20/21/5385/s1.

## Abbreviations

AChE	Acetylcholinesterase
BChE	Butyrylcholinesterase
PSII	Photosystem II
CAMD	Computer Assisted Molecular Design
ADMET	Absorption Distribution Metabolism Excretion Toxicity
CoMSA	Comparative Molecular Surface Analysis
SMV	Stochastic Model Validation
RIV	Rivastigmine
GLT	Galanthamine
DCMU	3-(3,4-dichlorophenyl)-1,1-dimethylurea
PQ	Plastoquinone
PCA	Principal Component Analysis
IVE-PLS	Iterative Variable Elimination Partial Least Squares
PLIP	Protein Ligand Interaction Profiler
PET	Photosynthetic Electron Transport
