# Supplementary material for: SAR-mediated Similarity Assessment of the Property Profile for New, Silicon-Based AChE/BChE Inhibitors
Source: Int J Mol Sci. 2019 Oct 29;20(21):5385. doi: 10.3390/ijms20215385 (PMC6862691; doi:10.3390/ijms20215385)
Supplement: Supplementary file 1 [file ijms-20-05385-s001.pdf]

Supplementary Materials

SAR-mediated Similarity Assessment of Property Profile for New Silicon-based AChE/BChE Inhibitors

Andrzej Bak [1,*], Hana Pizova [2,*], Violetta Kozik [1], Katarina Vorcakova [3], Jiri Kos [4], Jakub Treml [5], Klara Odehnalova [2], Michal Oravec [6], Ales Imramovsky [7], Pavel Bobal [2], Adam Smolinski [8], Zdeněk Trávníček [4] and Josef Jampilek [4,*]

[1] Institute of Chemistry, University of Silesia, Szkolna 9, 40 007 Katowice, Poland; violetta.kozik@us.edu.pl (V.K.)
[2] Department of Chemical Drugs, Faculty of Pharmacy, University of Veterinary and Pharmaceutical Sciences, Palackeho 1, 612 42 Brno, Czech Republic; pavelbobal@yahoo.com (P.B.)
[3] Department of Biological and Biochemical Sciences, Faculty of Chemical Technology, University of Pardubice, Studentska 573, 532 10 Pardubice, Czech Republic; katarina.vorcakova@upce.cz (K.V.)
[4] Division of Biologically Active Complexes and Molecular Magnets, Regional Centre of Advanced Technologies and Materials, Faculty of Science, Palacky University, Slechtitelu 27, 783 71 Olomouc, Czech Republic, jiri.kos@upol.cz (J.K.); zdenek.travnicek@upol.cz (Z.T.)
[5] Department of Molecular Biology and Pharmaceutical Biotechnology, Faculty of Pharmacy, University of Veterinary and Pharmaceutical Sciences, Palackeho 1, 612 42 Brno, Czech Republic; tremlj@vfu.cz (J.T.)
[6] Global Change Research Institute CAS, Belidla 986/4a, 60300 Brno, Czech Republic; oravec.m@czechglobe.cz
[7] Institute of Organic Chemistry and Technology, Faculty of Chemical Technology, University of Pardubice, Studentska 573, 532 10 Pardubice, Czech Republic; ales.imramovsky@upce.cz (A.I.)
[8] Department of Energy Saving and Air Protection, Central Mining Institute, Plac Gwarkow 1, 40 166 Katowice, Poland; smolin@gig.katowice.pl (A.S.)
* Correspondence: andrzej.bak@us.edu.pl (A.B.); pizovah@gmail.com (H.P.); josef.jampilek@gmail.com (J.J.)

Table S1. Protocol about BBB permeation of rivastigmine predicted using ACD/Percepta 14.0.0. Predicted values – Blood-Brain Barrier transport (rodent).

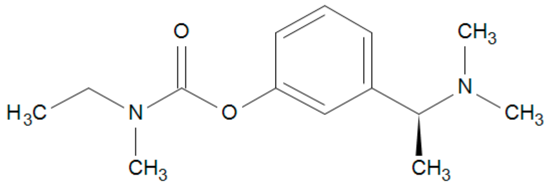

Main physico-chemical determinants:	
LogP	2.29
pKa (Acid)	No pKa
pKa (Base)	8.62
Fraction unbound in plasma	0.67

BBB transport parameters:	
LogPS	LogPS: -1.9
LogBB	LogBB: 0.51
Log(PS*fu, brain)	Log(PS*fu, brain): -2.6

Brain penetration sufficient for CNS activity.

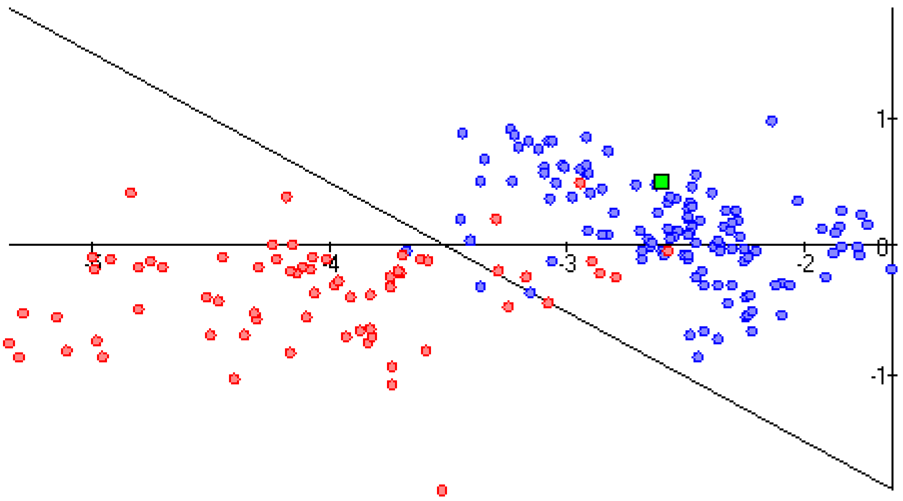

Table S2. Protocol about BBB permeation of galanthamine predicted using ACD/Percepta 14.0.0. Predicted values – Blood-Brain Barrier transport (rodent).

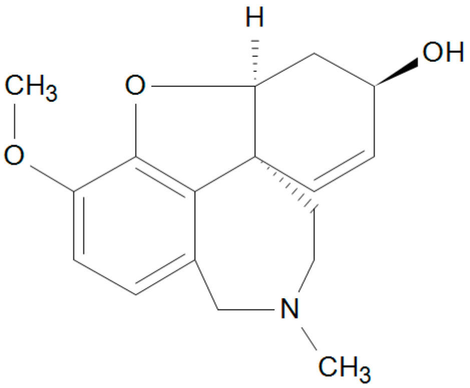

Main physico-chemical determinants:	
LogP	1.55
pKa (Acid)	13.93
pKa (Base)	7.92
Fraction unbound in plasma	0.80

BBB transport parameters:	
LogPS	LogPS: -2.2
LogBB	LogBB: 0.28
Log(PS*fu, brain)	Log(PS*fu, brain): -2.6

Brain penetration sufficient for CNS activity.
Carrier-mediated transport: - Organic cation transport. Typical for basic aromatic compounds (Vx < 2.5).

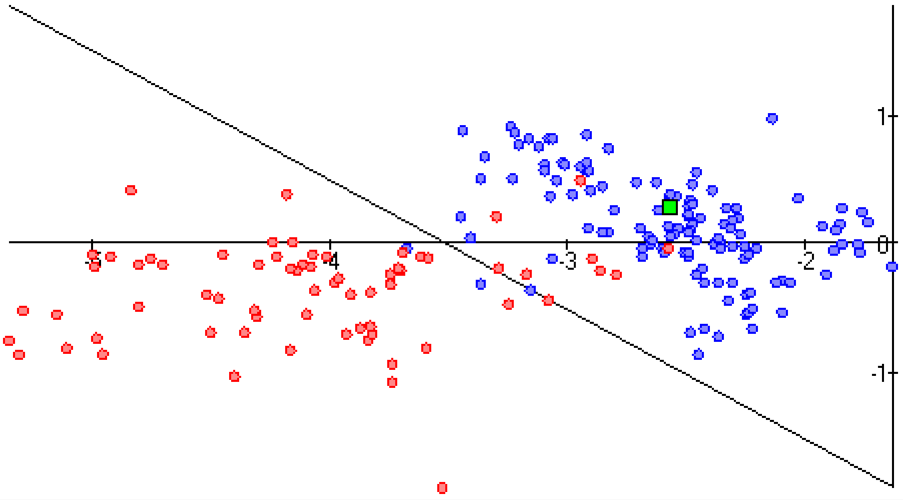

Table S3. Protocol about BBB permeation of donepezil predicted using ACD/Percepta 14.0.0. Predicted values – Blood-Brain Barrier transport (rodent).

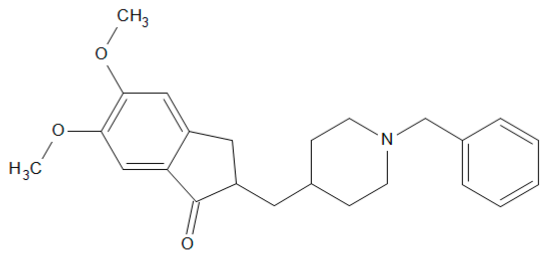

**Main physico-chemical determinants:**	
LogP	4.23
pKa (Acid)	No pKa
pKa (Base)	8.84
Fraction unbound in plasma	0.050

**BBB transport parameters:**	
LogPS	LogPS: -1.3
LogBB	LogBB: 0.41
Log(PS*fu, brain)	Log(PS*fu, brain): -3.1

Brain penetration sufficient for CNS activity.

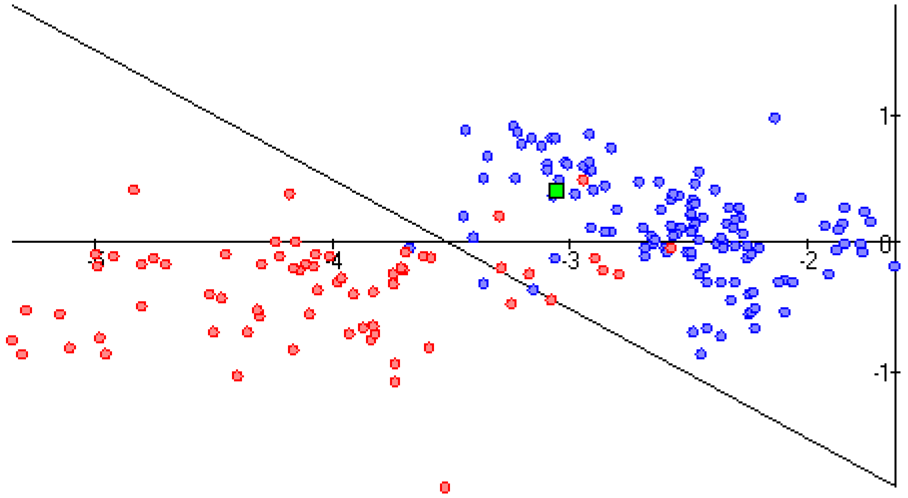

Table S4. Protocol about BBB permeation of tacrine predicted using ACD/Percepta 14.0.0. Predicted values – Blood-Brain Barrier transport (rodent).

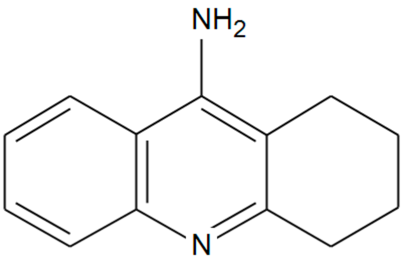

**Main physico-chemical determinants:**	
LogP	2.86
pKa (Acid)	No pKa
pKa (Base)	9.64
Fraction unbound in plasma	0.22

**BBB transport parameters:**	
LogPS	LogPS: -1.9
LogBB	LogBB: 0.30
Log(PS*fu, brain)	Log(PS*fu, brain): -2.9

Brain penetration sufficient for CNS activity.

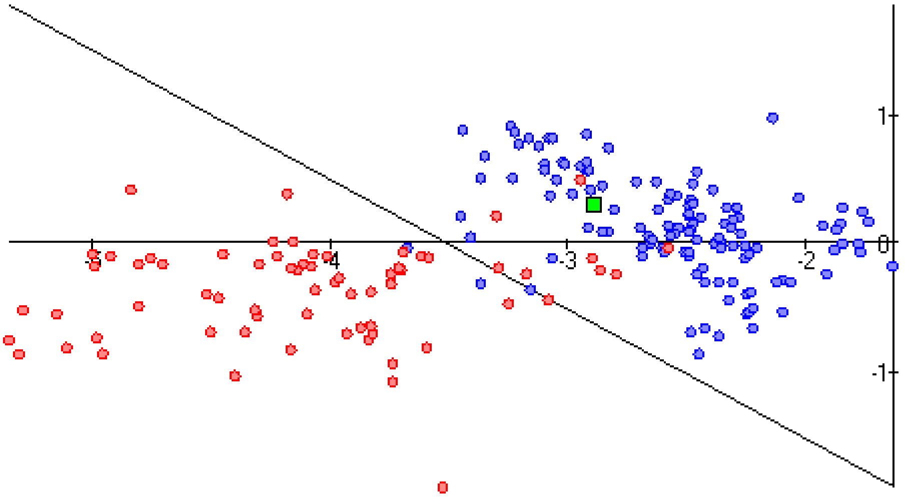

Table S5. Protocol about BBB permeation of Comp. No. **2** predicted using ACD/Percepta 14.0.0. Predicted values – Blood-Brain Barrier transport (rodent).

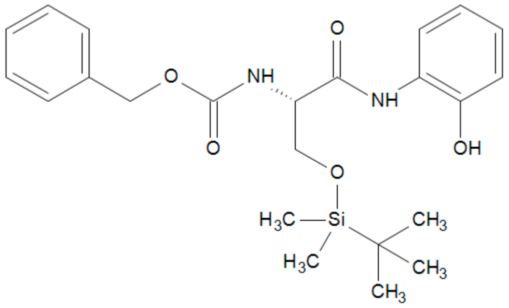

**Main physico-chemical determinants:**	
LogP	5.15
pKa (Acid)	9.32
pKa (Base)	1.86
Fraction unbound in plasma	0.11

**BBB transport parameters:**	
LogPS	LogPS: -1.4
LogBB	LogBB: 1.21
Log(PS*fu, brain)	Log(PS*fu, brain): -3.5

Brain penetration sufficient for CNS activity.

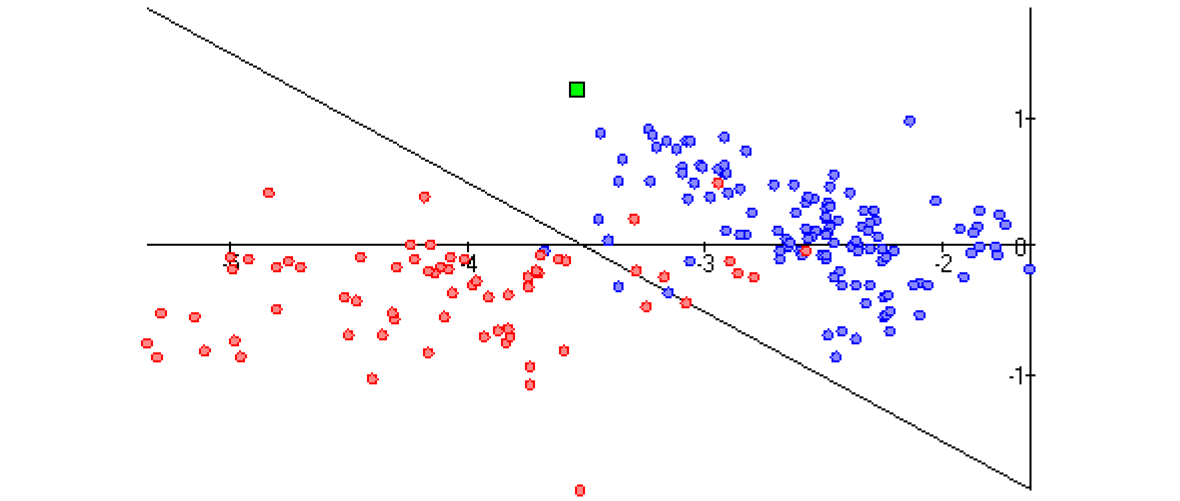

Table S6. Protocol about BBB permeation of Comp. No. **3** predicted using ACD/Percepta 14.0.0. Predicted values – Blood-Brain Barrier transport (rodent).

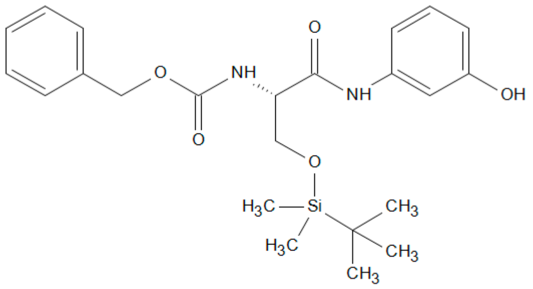

**Main physico-chemical determinants:**	
LogP	5.09
pKa (Acid)	9.48
pKa (Base)	0.12
Fraction unbound in plasma	0.076

**BBB transport parameters:**	
LogPS	LogPS: -1.4
LogBB	LogBB: 1.02
Log(PS*fu, brain)	Log(PS*fu, brain): -3.5

Brain penetration sufficient for CNS activity.

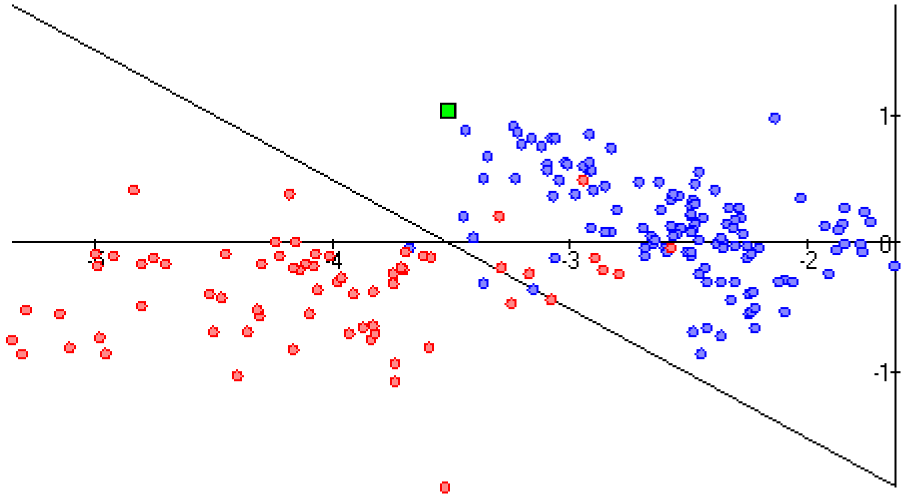

Table S7. Protocol about BBB permeation of Comp. No. **4** predicted using ACD/Percepta 14.0.0. Predicted values – Blood-Brain Barrier transport (rodent).

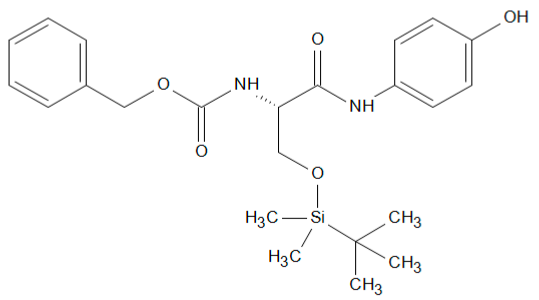

**Main physico-chemical determinants:**	
LogP	4.83
pKa (Acid)	9.99
pKa (Base)	1.59
Fraction unbound in plasma	0.082

**BBB transport parameters:**	
LogPS	LogPS: -1.4
LogBB	LogBB: 0.98
Log(PS*fu, brain)	Log(PS*fu, brain): -3.4

Brain penetration sufficient for CNS activity.

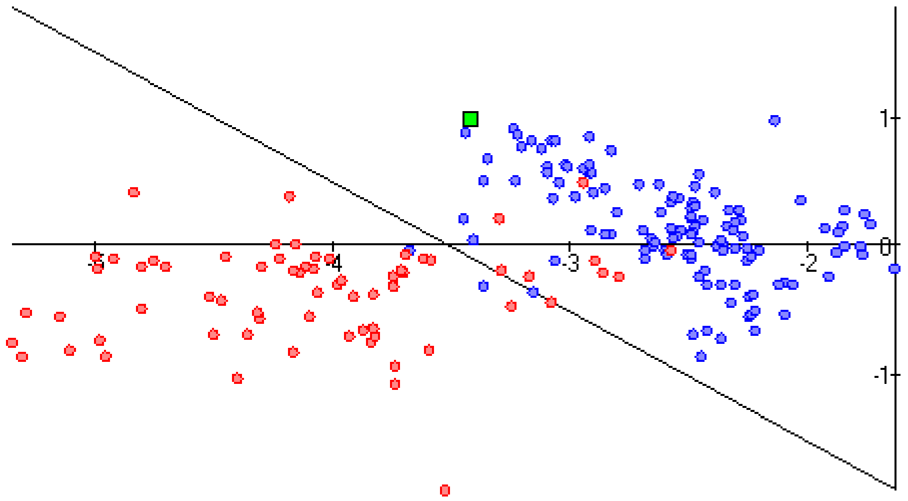

Table S8. Protocol about BBB permeation of Comp. No. **25** predicted using ACD/Percepta 14.0.0. Predicted values – Blood-Brain Barrier transport (rodent).

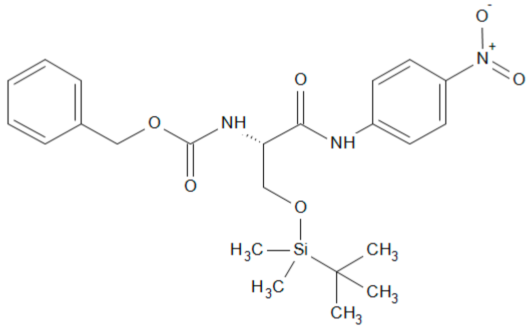

**Main physico-chemical determinants:**	
LogP	5.31
pKa (Acid)	10.48
pKa (Base)	-1.84
Fraction unbound in plasma	0.045

**BBB transport parameters:**	
LogPS	LogPS: -1.4
LogBB	LogBB: 0.73
Log(PS*fu, brain)	Log(PS*fu, brain): -3.5

Brain penetration sufficient for CNS activity.

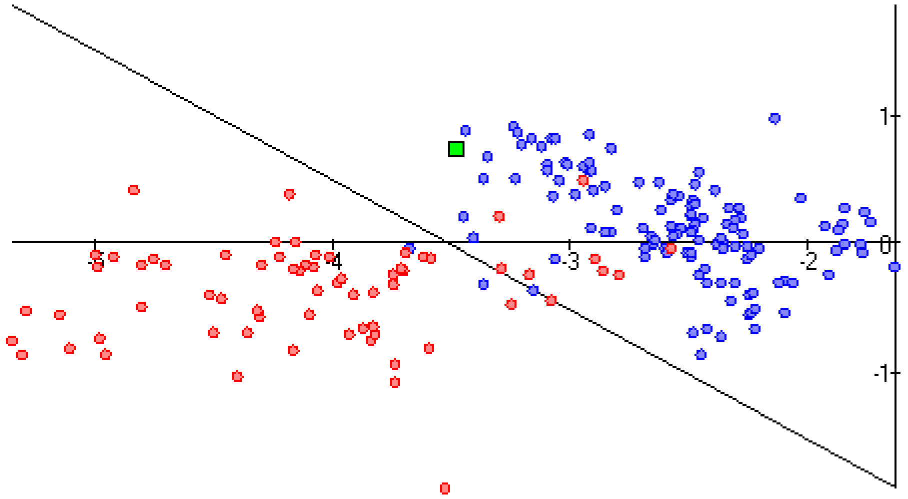

Table S9. Protocol about BBB permeation of Comp. No. **7** predicted using ACD/Percepta 14.0.0. Predicted values – Blood-Brain Barrier transport (rodent).

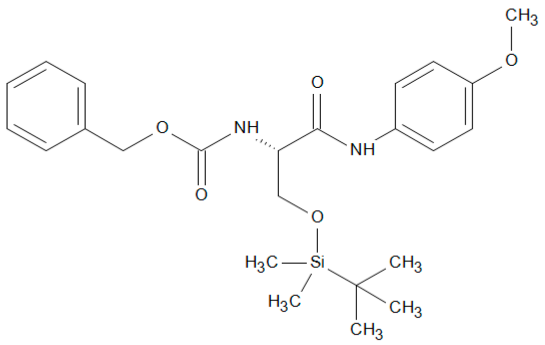

**Main physico-chemical determinants:**	
LogP	5.34
pKa (Acid)	10.60
pKa (Base)	1.23
Fraction unbound in plasma	0.070

**BBB transport parameters:**	
LogPS	LogPS: -1.3
LogBB	LogBB: 0.99
Log(PS*fu, brain)	Log(PS*fu, brain): -3.4

Brain penetration sufficient for CNS activity.

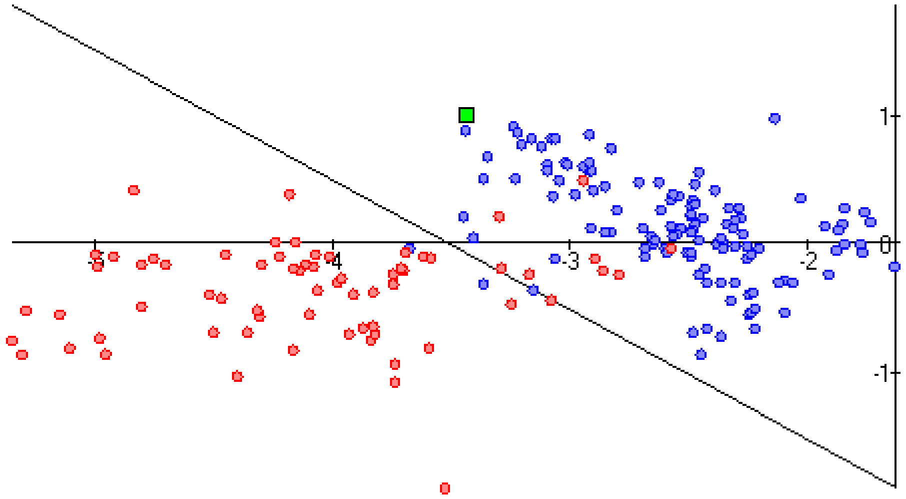

Table S10. Protocol about BBB permeation of Comp. No. **6** predicted using ACD/Percepta 14.0.0. Predicted values – Blood-Brain Barrier transport (rodent).

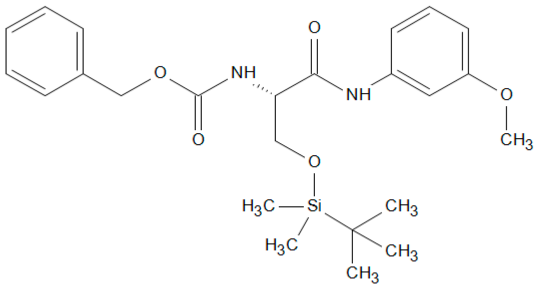

**Main physico-chemical determinants:**	
LogP	5.61
pKa (Acid)	10.58
pKa (Base)	0.12
Fraction unbound in plasma	0.057

**BBB transport parameters:**	
LogPS	LogPS: -1.4
LogBB	LogBB: 0.91
Log(PS*fu, brain)	Log(PS*fu, brain): -3.5

Brain penetration sufficient for CNS activity.

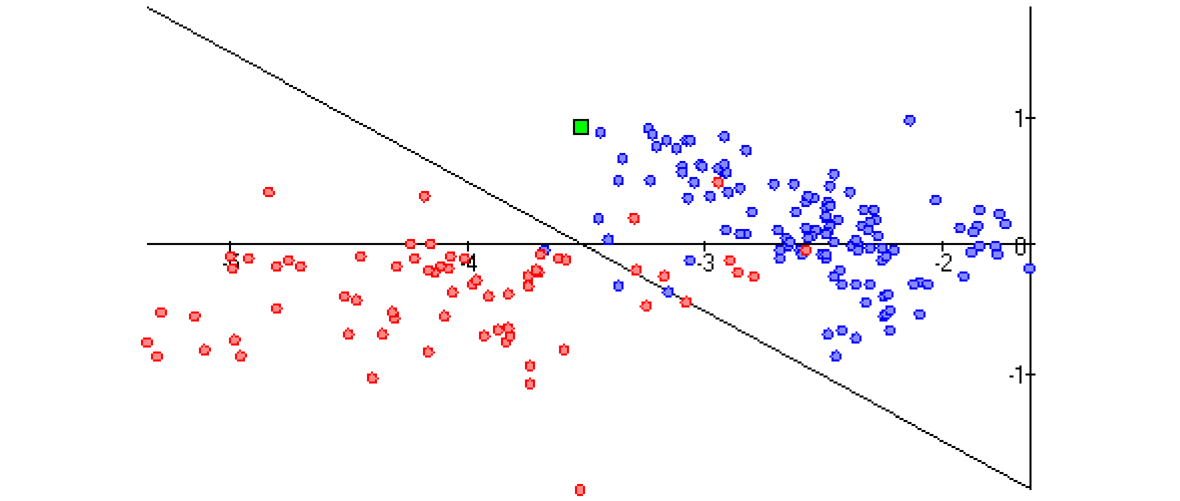

Table S11. Theoretically estimated partition coefficient calculated by set of alternative methods for *s*ilicon-based carbamates **1–25**.

No.	logP[a]	miLogP[b]	ClogP[c]	ClogP[d]	ClogP[e]	ClogP[f]	ClogP[g]	MlogP[h]	AlogP[i]	ClogP[j]	ClogP[k]
**1**	4.64	6.83	4.43	2.63	5.06	5.01	6.34	2.74	5.00	4.72	5.01
**2**	4.32	6.56	4.09	2.35	4.64	4.73	5.97	2.25	4.73	4.24	4.66
**3**	4.43	6.32	4.09	2.35	4.41	4.73	5.98	2.25	4.73	3.89	4.66
**4**	4.46	6.35	4.09	2.35	4.41	4.73	5.59	2.25	4.73	3.89	4.66
**5**	4.76	6.83	4.36	2.38	4.56	4.76	6.23	2.46	4.99	4.24	4.98
**6**	4.81	6.86	4.36	2.38	5.15	4.76	6.50	2.46	4.99	4.80	4.98
**7**	4.80	6.88	4.36	2.38	5.15	4.76	6.35	2.46	4.99	4.80	4.98
**8**	4.74	7.23	4.78	3.10	4.93	5.48	6.80	2.95	5.49	4.70	5.38
**9**	4.75	7.25	4.78	3.10	5.58	5.48	6.80	2.95	5.49	5.27	5.38
**10**	4.75	7.28	4.78	3.10	5.58	5.48	6.80	2.95	5.49	5.27	5.38
**11**	4.79	6.94	4.54	2.77	4.88	5.15	6.16	3.12	5.21	4.36	5.11
**12**	4.78	6.97	4.54	2.77	5.48	5.15	6.87	3.12	5.21	4.92	5.11
**13**	4.78	6.99	4.54	2.77	5.48	5.15	6.72	3.12	5.21	4.92	5.11
**14**	5.31	7.46	5.04	3.15	5.20	5.53	6.53	3.22	5.67	4.80	5.64
**15**	5.31	7.48	5.04	3.15	6.05	5.53	7.06	3.22	5.67	5.36	5.64
**16**	5.34	7.50	5.04	3.15	6.05	5.53	7.30	3.22	5.67	5.36	5.64
**17**	5.32	7.59	5.16	3.15	5.32	5.80	6.59	3.32	5.75	5.05	5.70
**18**	5.32	7.61	5.16	3.42	6.20	5.80	7.58	3.32	5.75	5.61	5.70
**19**	5.31	7.64	5.16	3.42	6.20	5.80	7.54	3.32	5.75	5.61	5.70
**20**	5.08	7.67	5.28	3.52	4.96	5.90	6.22	3.53	5.94	5.12	5.90
**21**	5.08	7.70	5.28	3.52	6.41	5.90	7.45	3.53	5.94	5.68	5.90
**22**	5.12	7.72	5.28	3.52	6.41	5.90	7.78	3.53	5.94	5.68	5.90
**23**	4.67	6.74	3.51	0.65	4.91	4.97	6.25	2.86	4.90	4.69	5.39
**24**	4.72	6.76	3.51	0.65	5.37	4.97	6.72	2.86	4.90	5.26	4.84
**25**	4.82	6.79	3.51	0.65	5.37	4.97	6.91	2.86	4.90	5.26	4.84

[a] clogPS, [b] Molinspirations, [c] OSIRIS property explorer, [d] HyperChem 7.0, [e] Sybyl X, [f] Marvin Sketch (ChemAxon) 15, [g] ChemSketch 2015, [h] Dragon 6.0, [i] Dragon 6.0, [j] Kowwin, [k] XlogP3.

Table S12. Matrix of correlation coefficients (n=25, α=0.05) of linear relationships between particular partition coefficients and experimental lipophilicity data (log *k*) for silicon-based carbamates **1–25**.

	**log *k***	**logP[a]**	**miLogP[b]**	**ClogP[c]**	**ClogP[d]**	**ClogP[e]**	**ClogP[f]**	**ClogP[g]**	**MlogP[h]**	**AlogP[i]**	**ClogP[j]**	**ClogP[k]**
**log*k***	1											
**logP[a]**	0.79	1										
**miLogP[b]**	0.90	0.89	1									
**ClogP[c]**	0.65	0.73	0.87	1								
**ClogP[d]**	0.41	0.52	0.70	0.95	1							
**ClogP[e]**	0.88	0.76	0.78	0.60	0.43	1						
**ClogP[f]**	0.87	0.81	0.97	0.85	0.69	0.73	1					
**ClogP[g]**	0.84	0.73	0.76	0.55	0.39	0.95	0.72	1				
**MlogP[h]**	0.90	0.80	0.90	0.71	0.52	0.75	0.92	0.73	1			
**AlogP[i]**	0.85	0.84	0.98	0.91	0.76	0.74	0.98	0.71	0.90	1		
**ClogP[j]**	0.91	0.75	0.81	0.52	0.31	0.93	0.76	0.91	0.79	0.74	1	
**ClogP[k]**	0.88	0.84	0.96	0.83	0.64	0.71	0.95	0.68	0.90	0.96	0.74	1

[a] clogPS, [b] Molinspirations, [c] OSIRIS property explorer, [d] HyperChem 7.0, [e] Sybyl X, [f] Marvin Sketch (ChemAxon) 15, [g] ChemSketch 2015, [h] Dragon 6.0, [i] Dragon 6.0, [j] Kowwin, [k] XlogP3.
